# LytTR Regulatory Systems: A potential new class of prokaryotic sensory system

**DOI:** 10.1371/journal.pgen.1007709

**Published:** 2018-10-08

**Authors:** Zhengzhong Zou, Hua Qin, Amanda E. Brenner, Rahul Raghavan, Jess A. Millar, Qiang Gu, Zhoujie Xie, Jens Kreth, Justin Merritt

**Affiliations:** 1 Department of Restorative Dentistry, School of Dentistry, Oregon Health and Science University, Portland, Oregon, United States of America; 2 Department of Biology, Portland State University, Portland, Oregon, United States of America; 3 MOE Key Laboratory of Industrial Fermentation Microbiology, College of Biotechnology, Tianjin University of Science and Technology, Tianjin, China; 4 Department of Molecular Microbiology and Immunology, Oregon Health and Science University, Portland, Oregon, United States of America; The University of Texas Health Science Center at Houston, UNITED STATES

## Abstract

The most commonly studied prokaryotic sensory signal transduction systems include the one-component systems, phosphosignaling systems, extracytoplasmic function (ECF) sigma factor systems, and the various types of second messenger systems. Recently, we described the regulatory role of two separate sensory systems in *Streptococcus mutans* that jointly control bacteriocin gene expression, natural competence development, as well as a cell death pathway, yet they do not function via any of the currently recognized signal transduction paradigms. These systems, which we refer to as LytTR Regulatory Systems (LRS), minimally consist of two proteins, a transcription regulator from the LytTR Family and a transmembrane protein inhibitor of this transcription regulator. Here, we provide evidence suggesting that LRS are a unique uncharacterized class of prokaryotic sensory system. LRS exist in a basal inactive state. However, when LRS membrane inhibitor proteins are inactivated, an autoregulatory positive feedback loop is triggered due to LRS regulator protein interactions with direct repeat sequences located just upstream of the -35 sequences of LRS operon promoters. Uncharacterized LRS operons are widely encoded by a vast array of Gram positive and Gram negative bacteria as well as some archaea. These operons also contain unique direct repeat sequences immediately upstream of their operon promoters indicating that positive feedback autoregulation is a globally conserved feature of LRS. Despite the surprisingly widespread occurrence of LRS operons, the only characterized examples are those of *S*. *mutans*. Therefore, the current study provides a useful roadmap to investigate LRS function in the numerous other LRS-encoding organisms.

## Author summary

The ability to sense stimuli triggered by the extracellular environment is a fundamental requirement of all cellular life. For prokaryotes, there are a variety of recognized classes of sensory systems that are used to detect and respond to environmental stimuli. In the current study, we provide the first evidence for the existence of a potentially new class of prokaryotic sensory system, which we refer to as LytTR Regulatory Systems (LRS). Here, we show that LRS are broadly distributed among prokaryotes and are distinct from the other commonly studied sensory systems like two-component signal transduction systems and ECF sigma factor systems. Presently, there are only two characterized examples of LRS, both from *Streptococcus mutans*. We employ these LRS as models to first define the key features of LRS and then demonstrate how some of these characteristics are likely universally conserved among the plethora of uncharacterized LRS in other organisms. Based upon these data, we further describe how these sensory systems are likely to function in diverse species and illustrate how to identify and investigate the function of novel LRS.

## Introduction

The capacity of bacteria to sense and respond to stimuli triggered by the extracellular environment is fundamental for survival, particularly in highly dynamic and/or competitive niches. Prokaryotes currently have several recognized classes of sensory signal transduction systems that are used specifically for this purpose. The most diverse class consists of the one-component systems, which contain single protein fusions of a signal-sensing input domain and a transcription regulatory output domain [1]. The vast majority of one-component systems are soluble proteins that utilize a diverse array of small molecules to modulate their transcription factor activity [1]. Among the best characterized classes of prokaryotic sensory systems are the phosphosignaling systems, exemplified by two-component signal transduction systems (TCSTS) and eukaryotic-like serine-threonine kinases/phosphatases (eSTK/P). Phosphosignaling systems respond to environmental stimuli using sensor proteins containing integrated kinase/phosphatase domains, which alter the phosphorylation status of downstream proteins involved in the signaling pathway. For TCSTS, phosphorylation typically controls the sequence-specific DNA binding affinity of one or more cognate transcription regulators [2–5], whereas eSTK/P usually regulate the phosphorylation status of a broad assortment of proteins [6–8]. The next major class of prokaryotic sensory systems is the extracytoplasmic function (ECF) sigma (σ) factors. Unlike TCSTS and eSTK/P, ECF systems do not typically encode enzymatic domains within sensor proteins; rather, gene expression is regulated through the production of alternative σ factors that dictate the promoter affinity of RNA polymerase [9, 10]. ECF σ factors are normally maintained in an inactive state through direct interactions with cotranscribed cognate anti-σ factors that are typically embedded within the cell membrane [11, 12]. ECF systems can be classified into 50 distinct subgroups [13, 14] and are activated when the anti-σ factor is inhibited via regulated proteolysis, protein-protein interactions, or through a signal-induced conformational change, thus liberating the σ factor to assemble within the RNA polymerase holoenzyme [15]. Finally, bacteria (and many other organisms) also utilize a variety of purine-derived second messenger systems to transduce sensory information via molecules such as cAMP, (p)ppGpp, cyclic di-GMP (c-di-GMP), cyclic di-AMP (c-di-AMP), and cyclic GMP-AMP (c-GAMP) [16]. With the exception of (p)ppGpp, these second messenger systems are generally regulated through the action of two classes of proteins: cyclases that create the second messengers and the phosphodiesterases that degrade them [16–21]. For (p)ppGpp, its synthesis is catalyzed by RelA-SpoT family enzymes [22]. Once created, these second messengers can bind directly to their target proteins or RNAs to modulate their functions [20, 23, 24].

Recently, we have been examining the regulatory function of two related signal transduction systems in *Streptococcus mutans*, which we previously named HdrRM and BrsRM. Both systems share a variety of features and appear to be distinct from the aforementioned signal transduction system paradigms. Homologs of these two *S*. *mutans* systems, which we broadly refer to as LytTR Regulatory Systems (LRS), can be found in various bacteria, particularly within the Firmicutes phylum [25]. Despite their widespread distribution, all putative LRS in other organisms remain uncharacterized. Thus, our current knowledge of LRS is presently limited to our previous studies of the HdrRM and BrsRM LRS [25–29]. These two LRS are both arranged within 2-gene operons with the first gene encoding a transcription regulator from the LytTR Family [30] and the adjacent downstream gene encoding a transmembrane protein inhibitor of the LRS regulator [25]. Under normal laboratory growth conditions, the HdrRM and BrsRM LRS are both maintained in a basal inactive state, due to the function of their cognate membrane inhibitor proteins [26, 27, 29]. Thus, the membrane proteins presumably serve as the proximal switches responsible for LRS activation, much like the analogous role of two-component system sensor kinases or ECF system anti-σ proteins. By mutating either of the membrane inhibitors HdrM or BrsM, it is possible to forcibly activate both LRS and examine their effect upon downstream gene expression. Surprisingly, the HdrRM and BrsRM LRS both contain largely overlapping regulons, which includes natural competence and bacteriocin genes in addition to both LRS operons [26–29]. Thus, these two LRS appear to be both autoregulatory and coregulatory. Furthermore, activation of bacteriocin gene expression by the LRS regulators HdrR and BrsR is critically dependent upon their interaction with direct repeat sequences found upstream of the bacteriocin gene promoters [27, 29]. These direct repeat sequences conform to a broadly defined consensus recognized by members of the LytTR Family [29, 30]. While the actual signals responsible for HdrRM and BrsRM activation are currently unknown, both LRS operons are induced by a rapid switch to high cell density growth conditions [26]. Intriguingly, HdrRM and BrsRM also jointly control a potent suicide-like cell death pathway, which underscores their potential ecological significance for *S*. *mutans* and perhaps other species [29]. Overall, it is clear that the HdrRM and BrsRM LRS are not cryptic regulators, rather they control distinct regulons that are integrated into a variety of genetic networks. In the current study, we sought to define the key characteristics and global distribution of LRS. We provide evidence that HdrRM, BrsRM, and several other previously unrecognized *S*. *mutans* LRS are actually members of a large family of analogous regulatory systems found amongst both bacteria and archaea. The conserved features of these systems indicate that LRS may comprise a previously unrecognized class of prokaryotic signal transduction system.

## Results

### Novel *S*. *mutans* LRS share key characteristics and are part of the core *S*. *mutans* genome

**Fig 1:**
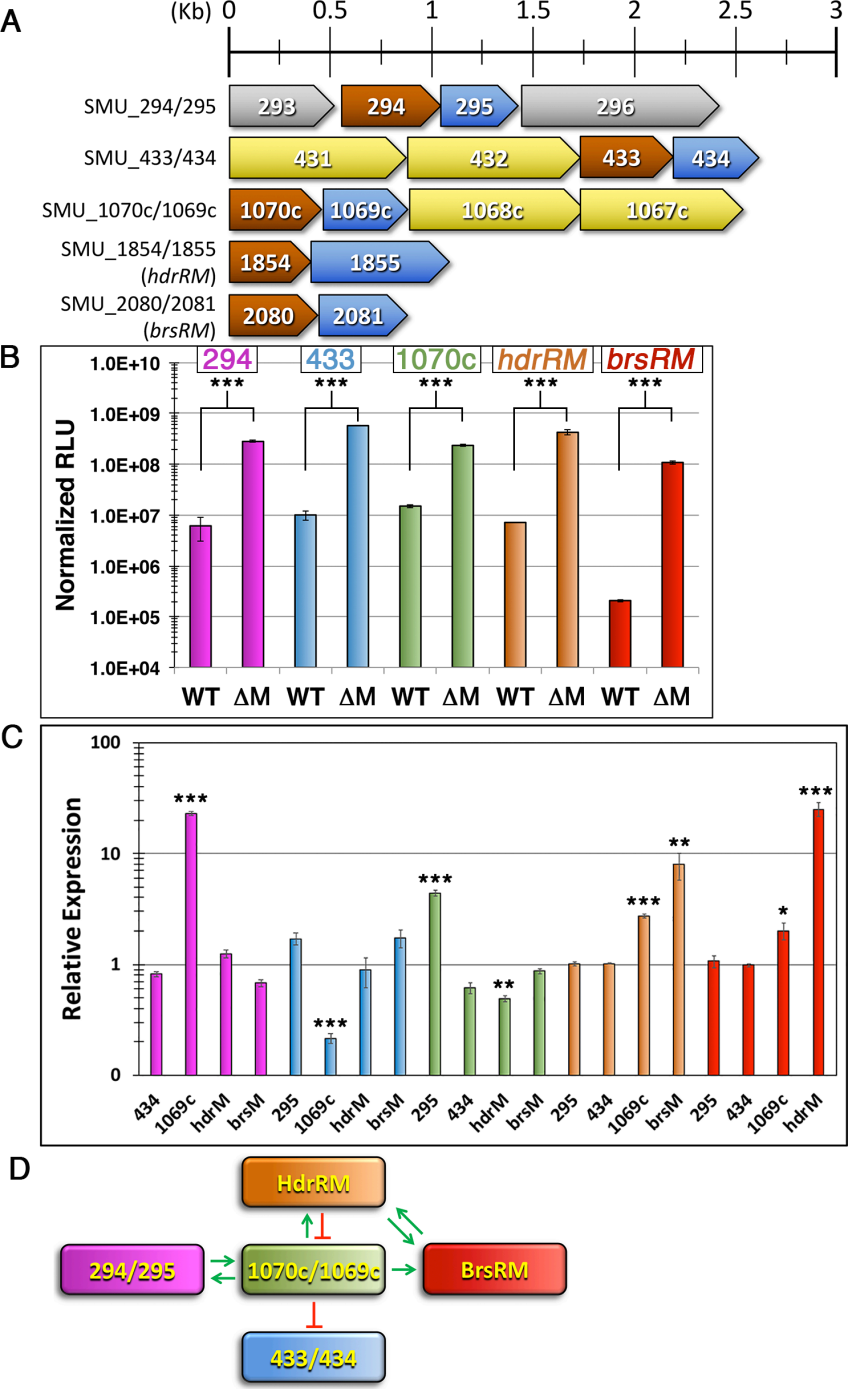
*S*. *mutans* encodes at least five sets of autoregulatory LRS. A) Schematic representation of the putative LRS encoded by *S*. *mutans*. Open reading frames are drawn to scale and color-coded as follows: LytTR Family regulator (brown), LRS membrane protein (blue), ABC transporter (yellow), other ORFs (grey). ORFs are numbered according to the terminal portions of their respective NCBI Gene Locus Tags (SMU_xxxx). B) Luciferase ORFs were inserted immediately downstream of each putative LRS to create transcription fusion reporters. Luciferase activity was normalized using optical density (OD_600_) and then compared between the wild-type (WT) and LRS membrane protein mutant reporter strains (ΔM). Data for each of the LRS reporter strains are color-coded as follows: SMU_294/295 (purple), SMU_433/434 (blue), SMU_1070c/1069c (green), HdrRM (orange), and BrsRM (red). C) Each of the putative LRS was tested pairwise for potential cross-regulation of other LRS operons. Luciferase activity of the mutant reporter strains was normalized to optical density (OD_600_) and then expressed relative to the parental reporter strain values, which were arbitrarily assigned values of 1. LRS reporter strains are color-coded as follows: SMU_294/295 (purple), SMU_433/434 (blue), SMU_1070c/1069c (green), HdrRM (orange), and BrsRM (red). Genes mutated in each of these reporter backgrounds are listed beneath each corresponding column. Unnamed genes are listed by the terminal portions of their respective NCBI Gene Locus Tags (SMU_xxxx). Statistical significance was assessed for each of the reporter strains exhibiting ≥2-fold difference in reporter activity relative to its parental reporter. D) Summary of cross-regulatory interactions between all five LRS. All luciferase data are expressed as means ± s.d. (indicated by error bars) derived from three or four biological replicates. ***P<0.001, **P<0.01, *P<0.05 Unpaired two-tailed Student’s *t*-test with Welch’s correction.

Our previous investigations of *S*. *mutans* LRS have focused upon the HdrRM and BrsRM LRS. However, it was unclear whether additional uncharacterized LRS might also exist in this species. Therefore, we began by searching the *S*. *mutans* genome for all of the transcription regulators containing putative LytTR Family DNA binding domains, which identified a total of seven genes. Two of these are obvious TCSTS response regulators (ComE and LytR), two are known LRS regulators (HdrR and BrsR), and the remaining three are uncharacterized hypothetical genes (SMU_294, SMU_433, and SMU_1070c). Inspection of the three uncharacterized genes revealed that all are arranged in apparent polycistronic operons and are upstream of open reading frames (ORFs) encoding putative transmembrane proteins (Fig 1A). This is highly reminiscent of the *hdrRM* and *brsRM* LRS operons, except that each of the uncharacterized operons also includes additional ORFs that are likely cotranscribed, whereas the *hdrRM* and *brsRM* operons are simply 2-gene operons. The SMU_294/295 genes are located between a conserved hypothetical gene (SMU_293) and an ORF encoding a putative ketopantoate reductase (SMU_296), while the SMU_433/434 and SMU_1070c/1069c genes are both likely cotranscribed with ABC transporter genes (Fig 1A). A key feature of the HdrRM and BrsRM LRS is their autoregulatory ability, which can be activated by mutagenesis of their respective membrane inhibitor proteins [26, 28, 29]. As shown in Fig 1B, each of the putative membrane proteins from all five operons was required to repress transcription of their respective operons indicating that the membrane proteins all similarly serve as inhibitors of an endogenous autoregulatory ability. The levels of induction triggered by the membrane protein deletions did vary widely however, with the SMU_294/295, SMU_433/434, and *hdrRM* operons all exhibiting ~50 to 60-fold maximum induction, while the SMU_1070c/1069c and *brsRM* operons exhibited <20-fold and >500-fold induction, respectively (Fig 1B). Overall, the expression characteristics of the operons were quite similar, except for the *brsRM* LRS, which has only a slightly lower maximum expression but a substantially lower basal expression. Thus, the dynamic range of inducibility for each of these operons seems primarily dependent upon the stringency of operon repression, rather than its maximum expression. In our previous studies, we also observed cross-regulation between the HdrRM and BrsRM LRS [28, 29]. Thus, we were interested to determine whether this is a unique feature of the HdrRM and BrsRM LRS or if other LRS might also exhibit cross-regulation of other LRS operons. To test this, we mutated each LRS membrane inhibitor protein and examined its resulting impact upon the other four non-cognate LRS luciferase reporter strains. To simplify the analysis, we deleted all but the two LRS of interest for each reporter to test every pairwise combination of LRS. With the exception of the SMU_433/434 LRS, all other LRS were found to trigger ≥2-fold change in reporter activity for one or more non-cognate LRS operons (Fig 1C). Several cross-regulatory interactions were quite strong, such as the opposing roles of the SMU_1070c/1069c LRS as both a potent activator of SMU_294/295 LRS operon expression and as an inhibitor of SMU_433/434 LRS expression (Fig 1C and 1D). The SMU_1070c/1069c LRS was also found to be particularly promiscuous, as it is the lone LRS capable of regulating all other LRS operons (Fig 1D). From these results, we can conclude that the activation of one LRS can influence the production of another, possibly as part of a regulatory network to modulate the kinetics associated with non-cognate LRS activation and/or the control of non-cognate LRS regulons.

As mentioned previously, the regulatory function of the HdrRM and BrsRM LRS in *S*. *mutans* is strongly indicative that they are not simply cryptic regulators. In further support of this notion, we examined whether the five *S*. *mutans* LRS operons are likely to be components of its core genome. 25 randomly selected *S*. *mutans* genomes were examined for the presence of all five operons and indeed all were present in every strain examined (Table 1). It should be noted that there were four strains in which *brsM* was either not annotated or annotated as a pseudogene due to the presence of apparent frameshift mutations within a poly-A region near the 3’ of the *brsM* ORF (Table 1). If such a mutation were truly present in *brsM*, it should constitutively activate BrsR in these strains. There was also a single instance in which *hdrR* was simply not annotated, even though the complete ORF is present (Table 1).

**Table 1:** *S*. *mutans* LRS operons are part of the core genome.

Strain	SMU_294	SMU_295	SMU_433	SMU_434	SMU_1070c	SMU_1069c	SMU_1854 (HdrR)	SMU_1855 (HdrM)	SMU_2080 (BrsR)	SMU_2081 (BrsM)
GS-5	SMUGS5_RS01270	SMUGS5_RS01275	SMUGS5_RS01975	SMUGS5_RS01980	SMUGS5_RS04780	SMUGS5_RS04775	SMUGS5_08350	SMUGS5_08355	SMUGS5_RS09430	SMUGS5_RS09435*
NN2025	SMUNN2025_RS08570	SMUNN2025_RS08565	SmuNN2025_1529	SmuNN2025_1528	SMUNN2025_RS05005	SMUNN2025_RS05010	SmuNN2025_0284	SmuNN2025_0283	SMUNN2025_RS09370	SMUNN2025_RS09375
PKUSS-LG01	PLG01_00262	PLG01_00263	PLG01_RS0109205	PLG01_RS07225	PLG01_RS04590	PLG01_RS04595	PLG01_01702	PLG01_01703	PLG01_01917	PLG01_01918
NLML8	SMU88_07197	SMU88_07202	SMU88_RS06890	SMU88_RS06895	SMU88_RS04905	SMU88_RS04910	SMU88_00375	SMU88_00380	SMU88_RS05640	SMU88_RS05635
15VF2	SMU40_07796	SMU40_07791	SMU40_RS08320	SMU40_RS08315	SMU40_RS03775	SMU40_RS03770	SMU40_07636	SMU40_07641	SMU40_RS06735	SMU40_RS06730
NV1996	SMU77_09132	SMU77_09137	SMU77_RS04625	SMU77_RS04620	SMU77_RS08555	SMU77_RS08560	SMU77_07681	SMU77_07686	SMU77_RS03320	SMU77_RS03325
S1B	SMU102_05654	SMU102_05649	SMU102_RS03520	SMU102_RS03515	SMU102_RS07325	SMU102_RS07330	SMU102_09448	SMU102_09453	SMU102_RS05585	SMU102_RS05590
5SM3	SMU50_08366	SMU50_08361	SMU50_RS07800	SMU50_RS07795	SMU50_RS01165	SMU50_RS01170	SMU50_07866	SMU50_07861	SMU50_RS02940	SMU50_RS02935
NVAB	SMU53_09635	SMU53_09640	SMU53_RS05955	SMU53_RS05950	SMU53_01385	SMU53_01380	SMU53_RS00300	SMU53_RS00305	SMU53_RS06530	SMU53_RS06535
SF12	SMU105_RS01360	SMU105_RS01355	SMU105_RS07335	SMU105_RS07330	SMU105_RS08645	SMU105_RS08640	SMU105_RS00340	SMU105_RS00335	SMU105_07142	SMU105_07137
3SN1	SMU26_RS06535	SMU26_RS06540	SMU26_RS04770	SMU26_RS04775	SMU26_RS07145	SMU26_RS07140	SMU26_08772	SMU26_08767	SMU26_09654	not annotated*
R221	SMU107_RS02805	SMU107_RS02800	SMU107_RS06685	SMU107_RS06680	SMU107_RS01840	SMU107_RS01845	SMU107_RS01190	SMU107_RS01185	SMU107_00213	SMU107_00208
OMZ175	SMU109_RS09430	SMU109_RS09425	SMU109_RS02930	SMU109_RS02925	SMU109_RS08705	SMU109_RS08710	SMU109_07636	SMU109_07641	SMU109_RS01905	SMU109_RS01900*
M230	SMU108_RS03395	SMU108_RS03400	SMU108_RS00445	SMU108_RS00450	SMU108_RS08330	SMU108_RS08335	SMU108_RS04130	SMU108_RS04135	SMU108_RS02300	SMU108_RS02295
2VS1	SMU41_RS02780	SMU41_RS02785	SMU41_RS02285	SMU41_RS02290	SMU41_RS09195	SMU41_RS09190	SMU41_RS00710	SMU41_RS00715	SMU41_RS08205	SMU41_RS08210
24	SMU99_RS01810	SMU99_RS01805	SMU99_RS04370	SMU99_RS04365	SMU99_RS06740	SMU99_RS06745	SMU99_06753	SMU99_06748	SMU99_RS03985	SMU99_RS03990
NMT4863	SMU57_RS00640	SMU57_RS00635	SMU57_RS03960	SMU57_RS03955	SMU57_RS08545	SMU57_RS08540	SMU57_06768	SMU57_06773	SMU57_RS06355	SMU57_RS06360*
NLML1	SMU89_RS00770	SMU89_RS00775	SMU89_RS03240	SMU89_RS03245	SMU89_RS06550	SMU89_RS06555	SMU89_04914	SMU89_04919	SMU89_RS02570	SMU89_RS02575
N66	SMU76_08650	SMU76_08645	SMU76_RS00735	SMU76_RS00740	SMU76_RS06520	SMU76_RS06525	SMU76_RS00960	SMU76_RS00955	SMU76_00800	SMU76_00805
NFSM1	SMU68_RS00360	SMU68_RS00355	SMU68_RS04130	SMU68_RS04135	SMU68_RS01560	SMU68_RS01555	SMU68_RS09285	SMU68_RS09280	SMU68_RS03260	SMU68_RS03255
NLML5	SMU70_RS00900	SMU70_RS00895	SMU70_RS08165	SMU70_RS08160	SMU70_RS02155	SMU70_RS02150	SMU70_RS05295	SMU70_RS05300	SMU70_08278	SMU70_08283
SM4	SMU97_08152	SMU97_08157	SMU97_RS01095	SMU97_RS01100	SMU97_RS07460	SMU97_RS07455	SMU97_RS01120	SMU97_RS01115	SMU97_RS01790	SMU97_RS01785
LJ23	SMULJ23_1676	SMULJ23_1675	SMULJ23_RS07900	SMULJ23_RS07895	SMULJ23_RS05010	SMULJ23_RS05015	SMULJ23_0305	SMULJ23_0304	SMULJ23_1824	SMULJ23_1825
NG8	APQ13_RS06435	APQ13_RS06430	APQ13_RS05775	APQ13_RS05770	APQ13_RS02945	APQ13_RS02950	not annotated	APQ13_RS09135	APQ13_RS08050	APQ13_RS08045
SF1	SMU80_RS05505	SMU80_RS05500	SMU80_RS07445	SMU80_RS07440	SMU80_RS04625	SMU80_RS04630	SMU80_RS03035	SMU80_RS03030	SMU80_RS06900	SMU80_RS06905

Genes are listed by their NCBI Gene Locus Tag designations.

*Indicates a frameshift mutation is present

### LRS are autoregulatory due to a positive feedback loop encoded within their operon promoters

**Fig 2:**
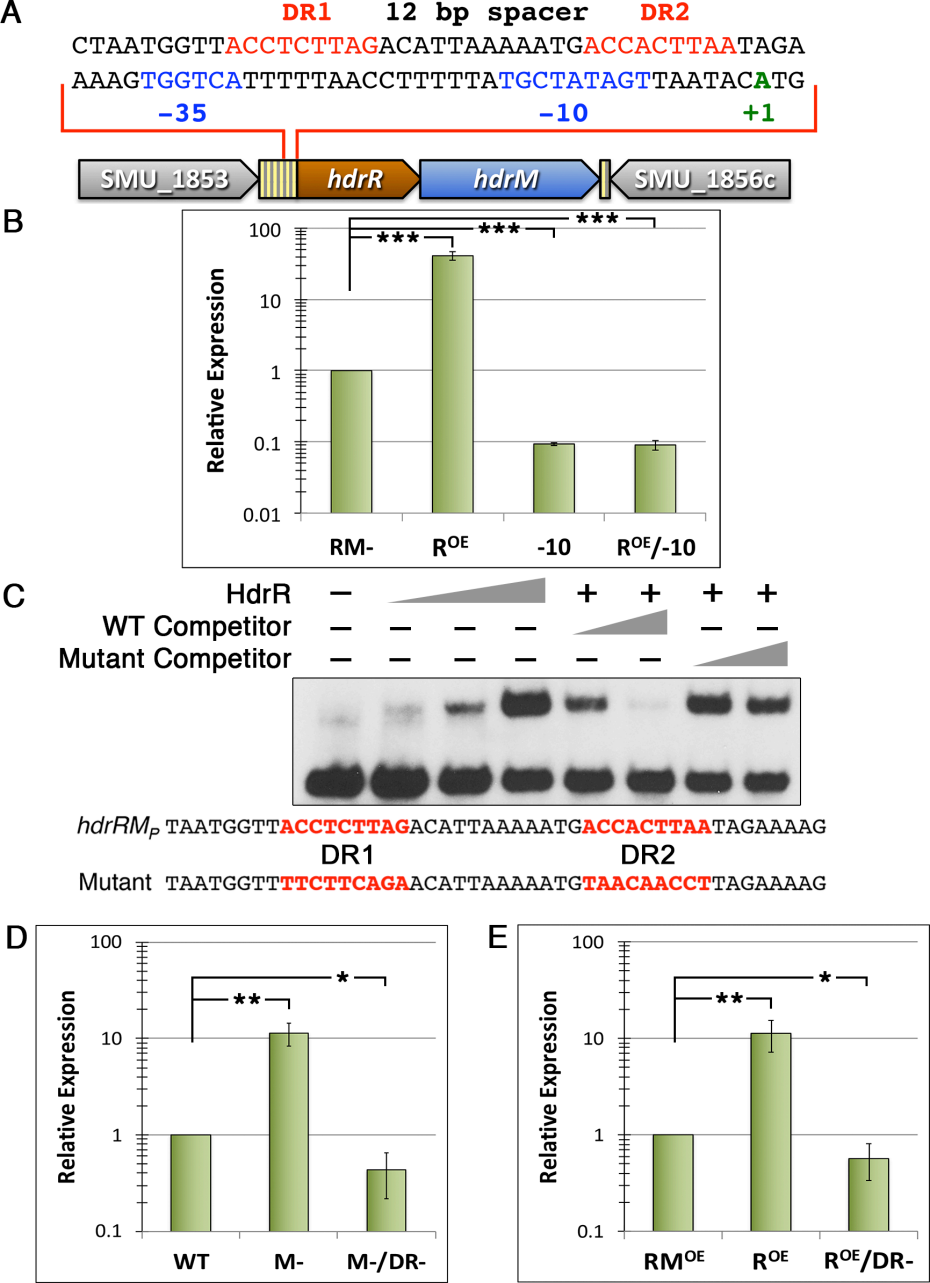
HdrRM operon regulatory elements mediate an autoregulatory positive feedback loop. A) Partial sequence of the intergenic region upstream of the *hdrRM* ORFs. The operon promoter is shown in blue font, the direct repeats are shown in red font, and the *hdrR* operon transcription start site (+1) is shown in green font. ORFs are shaded in solid colors, whereas intergenic regions are striped. B) An *hdrRM* luciferase reporter was created by replacing the *hdrRM* ORFs with that of luciferase. The luciferase activity of this parent reporter strain (RM-) was then compared after ectopic *hdrR* overexpression (R^OE^), mutation of the operon promoter -10 site (-10), and after combining *hdrR* ectopic overexpression with a mutant operon promoter -10 site (R^OE^/-10). Data are presented relative to the parent reporter strain, which was arbitrarily assigned a value of 1. C) Electrophoretic mobility shift assays (EMSAs) were performed with recombinant HdrR and 1 ng of a labeled DNA probe (*hdrRM*_*P*_) encompassing the direct repeat region upstream of the *hdrRM* operon promoter. To confirm the specificity of HdrR binding to the direct repeats, an unlabeled wild-type DNA probe (*hdrRM*_*P*_) and an unlabeled direct repeat mutant DNA probe (Mutant) were added to the EMSA reactions as competitors. The sequences of both competitor probes are presented under the EMSA image with the direct repeats shown in red. HdrR abundance per reaction: Lane 1 (0 μg), Lane 2 (10 μg), Lane 3 (20 μg), and Lanes 4–8 (30 μg). Wild-type competitor DNA (*hdrRM*_*P*_) abundance per reaction: Lane 5 (50 ng) and Lane 6 (200 ng). Mutant competitor DNA (Mutant) abundance per reaction: Lane 7 (50 ng) and Lane 8 (200 ng). D) An *hdrRM* luciferase reporter was created by placing a luciferase ORF immediately downstream of the *hdrRM* ORFs. The luciferase activity of this parent reporter strain (WT) was then compared after mutating *hdrM* (M-) and after doubly mutating *hdrM* and the operon direct repeats (M-/DR-). Data are presented relative to the parent reporter strain, which was arbitrarily assigned a value of 1. E) An *hdrRM* luciferase reporter was created by replacing the *hdrRM* ORFs with that of luciferase and then ectopically overexpressing *hdrR* in a single copy on the chromosome, while *hdrM* was ectopically expressed from a multicopy plasmid (i.e. uncoupled *hdrRM* expression). The luciferase activity of this reporter strain (RM^OE^) was then compared to an *hdrR* ectopic overexpression reporter strain (R^OE^) and an *hdrR* ectopic overexpression reporter strain with mutated operon direct repeats (R^OE^/DR-). Data are presented relative to the reporter strain RM^OE^, which was arbitrarily assigned a value of 1. All luciferase data are expressed as means ± s.d. (indicated by error bars) derived from four biological replicates. ***P<0.001, **P<0.01, and *P<0.05, Unpaired two-tailed Student’s *t*-test with Welch’s correction, significance compared to RM- (B), WT (D), and RM^OE^ (E).

Our previous transcriptomic analyses of the HdrRM and BrsRM LRS indicated that both systems are autoregulatory and coregulatory, as we observed potent induction of both LRS operons due to deletions of either of the LRS inhibitor proteins HdrM or BrsM [28, 29]. The same results could also be recapitulated via ectopic overexpression of either of the LRS regulator genes *hdrR* or *brsR* [28, 29]. As members of the LytTR Family of transcription regulators, both HdrR and BrsR would be predicted to recognize direct repeat sequences conforming to a broadly defined consensus [30]. Accordingly, LytTR Family consensus direct repeats are essential for HdrR and BrsR activation of bacteriocin gene expression [27, 29, 31–33]. However, a previous *in silico* analysis of the *S*. *mutans* genome failed to detect LytTR Family direct repeats in any of the LRS operon promoter regions [31]. Thus, we were curious whether the autoregulatory activity of LRS is mediated directly by the LRS regulators or via an indirect mechanism. As a test case, we first scanned the intergenic region upstream of the *hdrRM* operon to identify potential promoters. A strong candidate containing a putative extended -10 sequence was identified in this region in addition to a pair of direct repeats located 8 nucleotides upstream of the putative -35 sequence (Fig 2A). The spacing and length of the direct repeats are identical to those found in the multiple bacteriocin promoters regulated by HdrR and BrsR, but the operon direct repeat sequence diverges from the reported LytTR Family consensus [30–33]. This likely explains why it had not been previously detected. To further examine the identified operon promoter and direct repeats, we created two separate transcription fusion reporter strains, one in which a luciferase ORF replaced the *hdrRM* ORFs (i.e. Δ*hdrRM*) and another in which the luciferase ORF was inserted immediately downstream of the *hdrRM* ORFs (i.e. wild-type *hdrRM*). Using the Δ*hdrRM* reporter strain, we mutagenized the putative extended -10 sequence in the operon promoter, which resulted in substantially lower reporter activity compared to the parent strain (Fig 2B). In addition, the -10 deletion created a dominant phenotype that could not be suppressed even via ectopic *hdrR* overexpression, strongly supporting the role of this sequence as part of the operon promoter. To determine whether the upstream direct repeats might comprise an HdrR binding site, we performed electrophoretic mobility shift assays (EMSAs) using full-length recombinant HdrR and a small DNA fragment encompassing the *hdrRM* direct repeat region upstream of the -35. Sequence-specific mobility shifts were both detectable and critically dependent upon the identified direct repeats (Fig 2C). Next, we further assayed the same direct repeat mutations shown in Fig 2C using an *hdrRM* reporter strain containing a luciferase ORF inserted immediately downstream of the operon ORFs. A double mutation of *hdrM* and the direct repeats in this reporter confirmed that the direct repeat mutations are similarly dominant, as they resulted in reporter activity below that of the parent strain (Fig 2D). This indicated that the operon direct repeats further increase the basal expression of the operon via HdrR. It is worth noting that the basal luciferase activity of the Δ*hdrRM* reporter strain in Fig 2B is lower than that of the wild-type *hdrRM* reporter in Fig 2D (S1 Fig). We attributed this difference to modest levels of HdrR autoactivation upon the *hdrRM* operon promoter in the wild-type reporter strain and the lack of such regulation in the Δ*hdrRM* reporter. As further support for this notion, we created an ectopic *hdrRM* overexpression strain and observed an identical dependence upon the operon direct repeats to maintain the parental level of basal expression (Fig 2E). Thus, in addition to its role in bacteriocin production and natural competence development [27, 28], we can conclude that HdrR also directly serves as an autoregulatory transcription activator, triggering positive feedback autoregulation upon its own operon via two 9 bp direct repeat sequences located just upstream of the operon promoter. Next, we scanned the *brsRM* operon as well as the three other putative *S*. *mutans* LRS operons for similar promoter elements as those found in *hdrRM*. Like the *hdrRM* operon, we found that each of the other four operons indeed contain similar direct repeats located 4–11 bp upstream of their operon -35 sequences (Table 2). With the exception of the SMU_1070c/1069c LRS, each set of direct repeats is separated by 12 bp of intervening sequence. For the SMU_1070c/1069c LRS, the repeats are separated by 11 bp. For all five LRS, the locations of the direct repeats immediately upstream of the -35 sequences indicate they share similar regulatory mechanisms utilizing positive feedback autoactivation of their respective operons.

**Table 2:** *S*. *mutans* LRS all encode direct repeat-mediated autoregulation.

LytTR Regulator	Membrane Protein	Operon Direct Repeat	Spacer	Promoter
HdrR	HdrM	ACCTCTTAG-12 bp-ACCACTTAA	8 bp	TGGTCA-15 bp-TGCTATAGT
BrsR	BrsM	ACCACTTAT-12 bp-ACCGCTTAT	8 bp	TGGTTA-17 bp-TATACT
SMU_294	SMU_295	TCCTAGTAA-12 bp-TCCTTGTGT	4 bp	GCGACA-17 bp-TTTTAT
SMU_433	SMU_434	ACATCTTAT-12 bp-ACCTCTTAT	10 bp	GAGATT-14 bp-TGATAGACT
SMU_1070c	SMU_1069c	GCAACTTAG-11 bp-GCAACTTGA	11 bp	TTGTCA-13 bp-TGATATACT

### LRS are distinct from both TCSTS and ECF σ factor systems

**Fig 3:**
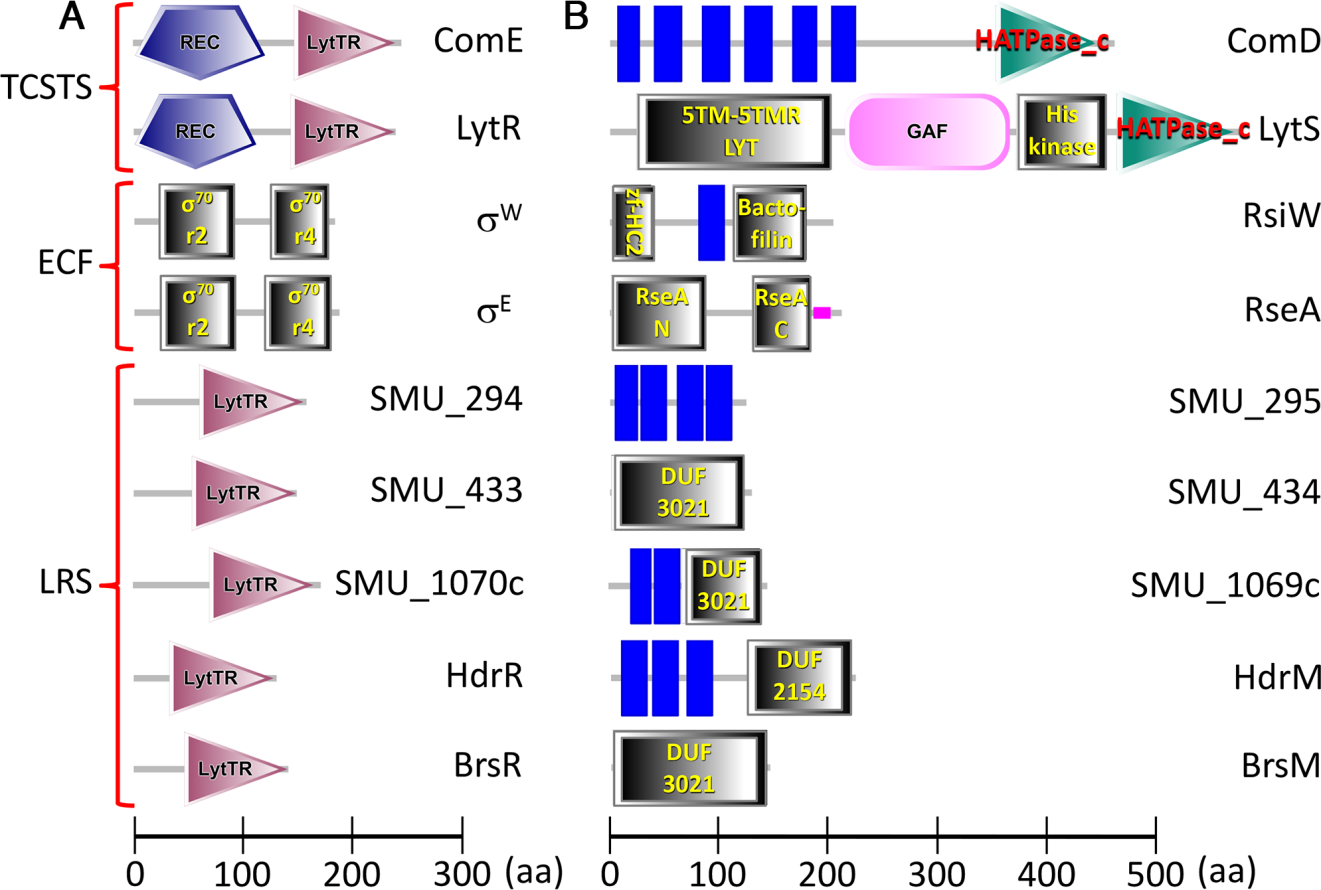
LRS are distinct from TCSTS and ECF systems. A) Comparison of the domain architectures of TCSTS response regulators, ECF σ factors, and LRS regulators. The illustrated proteins are from *S*. *mutans* with the exception of σ^W^ (*B*. *subtilis*) and σ^E^ (*E*. *coli*) and are all drawn to scale. Individual protein domains are labeled accordingly. B) Comparison of the domain architectures of TCSTS sensor kinases, ECF anti-σ factors, and LRS membrane proteins. The illustrated proteins are from *S*. *mutans* with the exception of RsiW (*B*. *subtilis*) and RseA (*E*. *coli*) and are all drawn to scale. Individual protein domains are labeled accordingly. Blue rectangles indicate transmembrane segments that are not located within identified conserved protein domains. All proteins were illustrated and annotated using the SMART webserver (http://smart.embl-heidelberg.de) [63].

LRS share some analogous features that are highly reminiscent of TCSTS and ECF σ factor systems. In fact, while searching for novel LRS operons in *S*. *mutans* and other species, we noticed a number of instances in which uncharacterized LRS regulators are erroneously annotated as LytTR Family response regulators. This would imply that such genes encode members of TCSTS, perhaps as orphan response regulators. While the LytTR Family does include numerous TCSTS response regulators, most members of this family are not [5, 30]. We compared the domain architectures of the two *S*. *mutans* response regulators containing LytTR Family DNA binding domains (ComE and LytR) with each of the five *S*. *mutans* LRS regulators. While the sizes of all of the LytTR Family DNA binding domains are comparable, the response regulators ComE and LytR are larger proteins overall due to the additional presence of signal receiver domains (Fig 3A), which are key features found in canonical response regulators [5] and are notably absent from the LRS regulators. Likewise, response regulators encode strictly conserved aspartate residues that are essential for phosphosignaling (S2A Fig), yet these are also absent from LRS regulators (S2B Fig). Obvious differences are similarly apparent when comparing TCSTS sensor kinases with LRS membrane inhibitors. The cognate sensor kinases for ComE and LytR (ComD and LytS, respectively) are considerably larger proteins due to the presence of various sensory domains and/or ATPase domains (Fig 3B), which are key features essential for sensor kinase function [34]. No predicted kinase domains or any other putative enzymatic functions are detectable in the five LRS membrane proteins, although four of these proteins do encode either of two Domains of Unknown Function (DUF3021 or DUF2154) (Fig 3B).

Like TCSTS, ECF σ factor systems are a major class of prokaryotic multi-protein sensory signal transduction system that share some analogous characteristics of LRS. One of the defining features of ECF systems is their utilization of ECF σ factors, which are distinct from those in the σ^70^ family, due to their lack of the conserved sigma 3 region (Fig 3A) [9, 35]. Both conserved domain analyses (Fig 3A) and DNA binding characteristics (Fig 2A–2E) clearly indicate that LRS regulators are bona fide transcription factors rather than σ factors, thus precluding them from being part of true ECF systems. Regardless, LRS membrane proteins do share some basic characteristics with most ECF anti-σ factors, as they are similarly sized membrane proteins, lack obvious enzymatic domains, and serve as inhibitors (Figs 1B and 3B) [11, 12]. Interestingly, after screening the genome sequence data of a phylogenetically diverse group of ECF system-encoding bacteria, we identified at least 10 separate Domains of Unknown Function encoded by ECF anti-σ factors, but we were unable to identify a single instance of anti-σ factors encoding either DUF3021 or DUF2154. Thus, this could be one major distinction between anti-σ factors and LRS membrane proteins.

### Key features of LRS are widely conserved among prokaryotes

**Fig 4:**
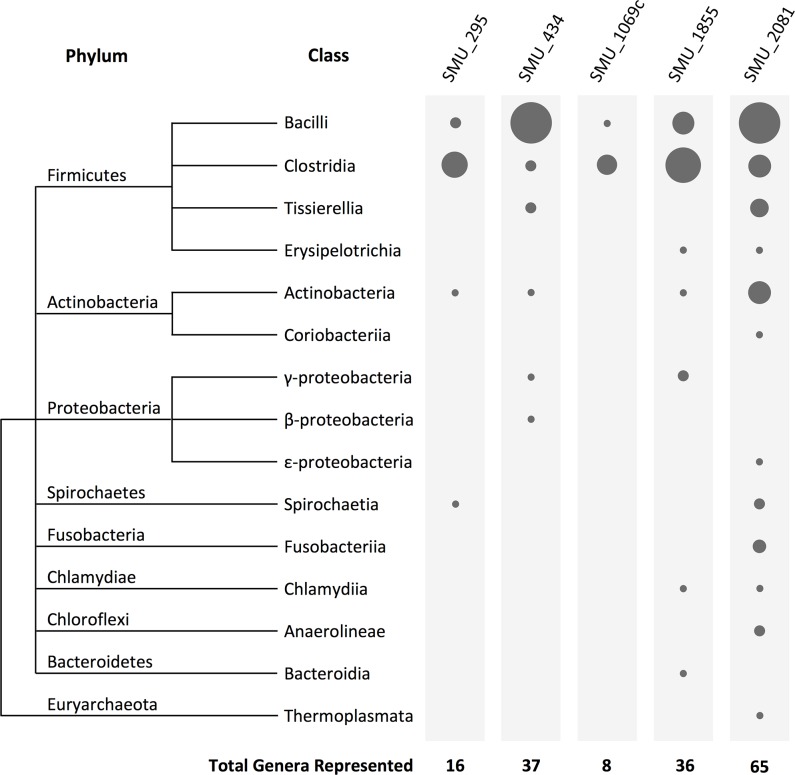
**Global distribution of putative LRS among prokaryotes.** The membrane proteins from the five *S*. *mutans* LRS were used as queries to identify potential LRS within the genome data of the NCBI non-redundant nucleotide collection (nr/nt) and whole genome shotgun (wgs) databases. The sizes of the filled circles are proportional to the number of identified genera encoding putative LRS matching to each of the corresponding *S*. *mutans*-type LRS.

**Fig 5:**
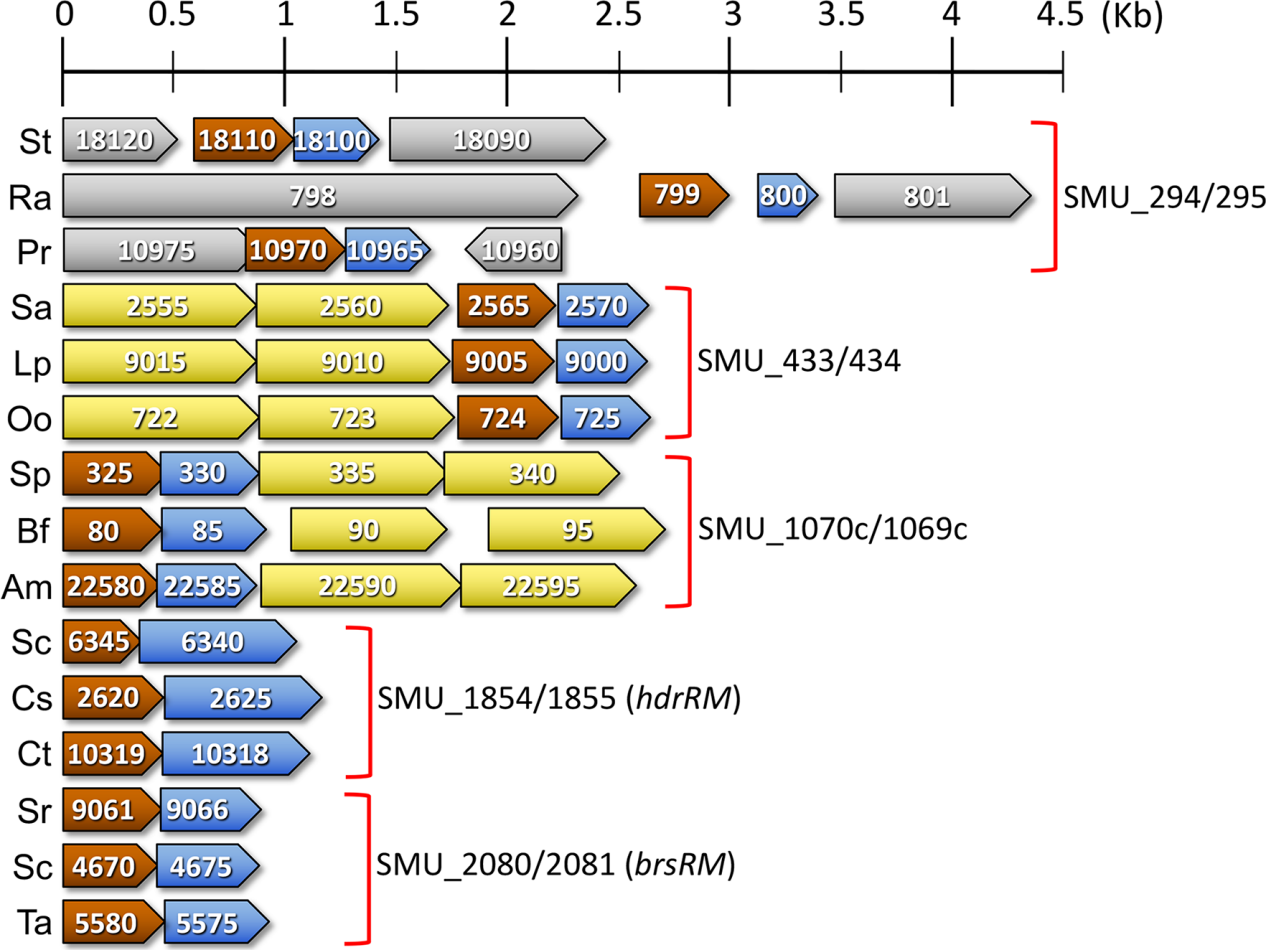
Comparison of LRS operons among diverse prokaryotes. A set of three representative operons matching to each of the five *S*. *mutans* LRS was randomly selected from the master table of putative LRS (S3 Table) and illustrated for comparison. In each set of three operons, their descending order in the figure is indicative of their relative homologies to the corresponding *S*. *mutans* LRS (Top = high homology, middle = medium homology, and bottom = low homology). Open reading frames are drawn to scale and color-coded as follows: LytTR Family regulator (brown), LRS membrane protein (blue), ABC transporter (yellow), and other ORFs (grey). ORFs are numbered according to the terminal portions of their respective NCBI Gene Locus Tags. Species and Gene Locus Tags are listed from top to bottom as: St (*Streptococcus troglodytae*; SRT_xxxxx), Ra (*Rothia aeria*; RA11412_0xxx), Pr (*Pseudobutyrivibrio ruminis*; CSX00_RSxxxxx), Sa (*Streptococcus anginosus*; SAIN_RS0xxxx), Lp (*Lactobacillus plantarum*; LPST_RS0xxxx), Oo (*Oenococcus oeni*; OEOE_0xxx), Sp (*Streptococcus pantholopis*; A0O21_RS00xxx), Bf (*Butyrivibrio fibrisolvens*; G624_RS01100xx), Am (*Anaerosporobacter mobilis*; BUB90_RSxxxxx), Sc (*Streptococcus caviae*; BMI76_0xxxx), Cs (*Clostridium sciendens*; CLOSCI_RS0xxxx), Ct (*Chlamydia trachomatis*; ERS095036_xxxxx), Sr (*Streptococcus ratti*; SRA_0xxxx), Sc (*Staphylococcus carnosus*; VV61_0xxxx), and Ta (Thermoplasmatales archaeon; TALC_RS0xxxx).

Given the highly conserved features of *S*. *mutans* LRS operons, we expanded our search for putative LRS in other species and were surprised to discover that LRS are encoded by a far broader diversity of organisms than previously recognized (Fig 4 and S3 Table). Using a multi-tiered search strategy modeled on the five *S*. *mutans* LRS, we were able to identify >4600 putative LRS operons spread amongst the genomes of numerous Gram positive and Gram negative bacteria as well as some archaea (S3 Table). Overall, the majority of identified LRS are encoded within the Firmicutes phylum, which agrees with previous observations [25]. Of the five *S*. *mutans* LRS, the BrsRM-type LRS exhibits the most diverse distribution and is the most commonly encoded (Fig 4). In all cases, the identified LRS operons are arranged similarly as in *S*. *mutans* with the LRS regulator encoded upstream of the membrane inhibitor (S3 Table). We also observed a conservation of ABC transporter genes linked to the SMU_433/434-like and SMU_1070c/1069c-like LRS of other species (Fig 5). The conserved co-occurrence of LRS and ABC transporter genes suggests that the respective encoded proteins all function together in related genetic pathways. However, this was not the case for the genes surrounding the SMU_294/295-type LRS, as only the very closely related species *Streptococcus troglodytae* contained a similar 4-gene operon (Fig 5). Therefore, the 4-gene operon structure of the *S*. *mutans* SMU_294/295 LRS (Fig 1A) is presumably either incidental or a niche-specific adaptation. Intriguingly, the LRS operons of other organisms all share highly analogous promoter regions to those of *S*. *mutans* LRS indicating that they similarly function via positive feedback autoregulation. Table 3 illustrates some of the diversity of LRS operon promoter elements that can be identified in both bacteria and archaea. Similar to *S*. *mutans*, most LRS operon direct repeat sequences are separated by 12 bp, but a minority is separated by either 11 bp or 13 bp. It is also evident there is a particularly strong bias for the direct repeats to be oriented 10 bp upstream of -35 sequences. The *S*. *mutans* LRS operons are somewhat unusual in this regard, as only the SMU_433/434 LRS operon contains direct repeats located exactly 10 bp upstream of the operon promoter. We also used Protter [36, 37] to illustrate the predicted topologies of *S*. *mutans* LRS membrane proteins to their corresponding weakest homology examples shown in Fig 5 and all yielded highly similar structures despite their limited sequence similarities (S3A–S3E Fig). Overall, the data indicate that most of the identified LRS in S3 Table are highly likely to be orthologs of the *S*. *mutans* proteins. While searching for putative LRS in other species, we also encountered a number of potentially novel LRS-types that are not found in *S*. *mutans*. The LRS listed in Table 3 for *Staphylococcus aureus*, *Listeria monocytogenes*, and *Treponema bryantii* have characteristics that are all nearly identical to the LRS found in *S*. *mutans*, except that their LRS membrane proteins exhibit no obvious homology to those of *S*. *mutans*. For the *S*. *aureus* membrane protein SACOL_RS12400, its predicted topology is also obviously distinct from the five *S*. *mutans* LRS membrane proteins (S3F Fig). Furthermore, members of the *Bacteroides fragilis* group, such as *B*. *thetaiotaomicron* and *B*. *ovatus*, encode “LRS-like” operons (Btheta7330_RS19920/RS19915 and Bovatus_RS21370/RS21375) that exhibit a number of distinctions from *S*. *mutans* LRS. These *Bacteroides* operons encode the membrane proteins upstream of the LytTR Family regulators. Unlike *S*. *mutans* LRS, the encoded membrane proteins contain two conserved domains, an NfeD-like domain in addition to DUF2154, which is the same domain found in the *S*. *mutans* LRS membrane protein HdrM (Figs 3B and S3G). The LytTR Family regulators encoded in the *Bacteroides* operons are also unusual, as they contain multiple transmembrane segments before the DNA binding domains, whereas all of the *S*. *mutans*-type LRS encode soluble transcription regulators (Fig 3A). The intergenic regions of the *Bacteroides* LRS-like operons also contain 11 bp direct repeats separated by 11 bp of intervening sequence with the repeats located 11 bp upstream of the operon promoters [38, 39] (Table 3). Presumably, these repeats similarly function in autoregulatory transcription activation of the operons. The presence of these distinct LRS-like operons indicates that additional uncharacterized varieties of LRS are likely to exist.

**Table 3:** Autoregulatory LRS are encoded by diverse bacteria and archaea.

Species	LytTR Regulator	Membrane Protein	Operon Direct Repeat	Spacer	Promoter	Class
***S*. *mutans*-type LRS**						
*Streptococcus pneumoniae*	spr1731	spr1730	ACCACTTAC-12 bp-ACCACTTGC	10 bp	TTGTAT-14 bp-TGATATAGT	Bacilli
*Streptococcus anginosus*	SANR_RS01820	SANR_RS01825	ACCACTTAC-12 bp-ACCACTTAC	10 bp	TTGAAT-13 bp-TGTTATAAT	Bacilli
*Bacillus cereus*	BCERE0022_RS11195	BCERE0022_11190	ACCAGTTAT-12 bp-ACCGACTAT	10 bp	TTTACA-17 bp-TATATT	Bacilli
*Staphylococcus carnosus*	VV61_04670	VV61_04675	ACCGCTTGT-12bp-ACCGCTTAT	10 bp	TTCTTA-14 bp-TGGTTTAAT	Bacilli
*Clostridium botulinum*	CLL_RS22995	CLL_RS23000	ACCACTTAC-12 bp-ACCACTTAT	10 bp	TTTACA-17 bp-TATAAT	Clostridia
*Peptostreptococcus anaerobius*	HMPREF0631_RS05510	HMPREF0631_RS05505	ACCTCTTAT-12 bp-ACCTTTTGT	11 bp	TGCAGA-16 bp-TATAAT	Clostridia
*Roseburia intestinalis*	ARA28_RS17855	ARA28_RS17860	ACCACTTAC-13 bp-ACCACTTG	11 bp	TTTACA-17 bp-TATAAT	Clostridia
*Anaerococcus prevotii*	APRE_RS08445	APRE_RS08450	TCCACTTAT-12 bp-ACCTTTTAT	10 bp	GTGAAT-17 bp-TATAAT	Tissierellia
*Propionimicrobium lymphophilum*	G556_RS11075	G556_RS0106150	GCCAGCTTG-13 bp-ACCGCTTAG	10 bp	TTGTAC-16 bp-TTTAAC	Actinobacteria
*Varibaculum cambriense*	HMPREF1862_RS06695	HMPREF1862_RS06700	CCCGCTTGG-12 bp-GCCGCTTAG	10 bp	TTGTAC-16 bp-TTTAAC	Actinobacteria
*Actinomyces turicensis*	HMPREF9241_RS01025	HMPREF9241_RS01030	CCCGCTTGG-12 bp-ACCGCTTAG	10 bp	TTGTAC-16 bp-TTTAAC	Actinobacteria
*Corynebacerium uterequi*	CUTER_RS00040	CUTER_RS00035	ACCACTTAG-12 bp-ACCGCATAG	10 bp	TTGCTC-17 bp-TGTACT	Actinobacteria
*Chlamydia trachomatis*	ERS095036_10319	ERS095036_10318	ACCACTTAC-12 bp-ACCACTTAC	10 bp	TTGAAT-13 bp-TGTTATAAT	Chlamydiae
*Chlamydia trachomatis*	ERS133248_00994	ERS133248_00993	ACCGCTTAT-12 bp-ACCAGATAG	10 bp	TTGAGT-16 bp-TTTTAC	Chlamydiae
*Thermoplasmatales BRNA1*	TALC_RS05580	TALC_RS05575	TCCGTCGGT-11 bp-TACGAGGGA	11 bp	TTGTCC-16 bp-TATATG	Thermoplasmata
**Putative Novel LRS**						
*Staphylococcus aureus*	SACOL_RS11195	SACOL_RS12400	GCCACTTAA-12 bp-ACCATTCAA	9 bp	AATATA-14 bp-TGGTTTAAT	Bacilli
*Listeria monocytogenes*	lmo0984	lmo0985	GCATCTTAG-12 bp-GCATGTTAC	10 bp	TTGTAG-16 bp-TATAAT	Bacilli
*Treponema bryantii*	SAMN04487977_102124	SAMN04487977_102123	ACCACTTAT-11 bp-GCCACTTAT	10 bp	CACACA-17 bp-TATACT	Spirochaetes
*Bacteroides thetaiotaomicron*	Btheta7330_RS19915	Btheta7330_RS19920	TCCGGTATTCA-11bp-ACCGGAAATCA	11 bp	TGTA-19 bp-TATCTTTG	Bacteroidia
*Bacteroides ovatus*	Bovatus_RS21375	Bovatus_RS21370	TCCGGCATTCA-11 bp-ACCGTAAATCA	11 bp	TGTG-19 bp-TATCTTTG	Bacteroidia

### Activation of the BrsRM LRS is intimately connected with purine metabolism

**Fig 6:**
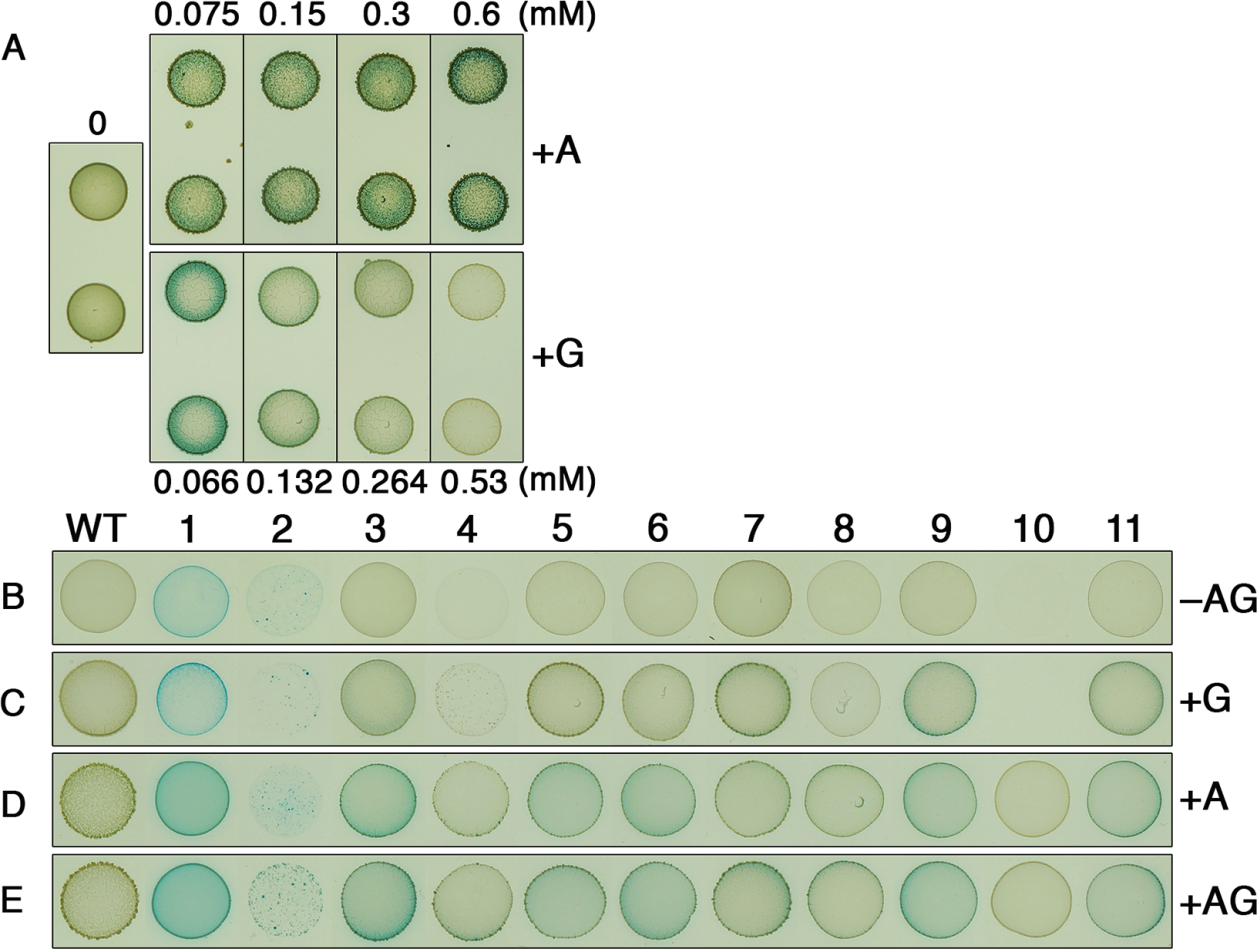
Purines mediate activation of the BrsRM LRS. A) The *brsRM-gusA* reporter was spotted onto chemically defined medium agar plates ± adenine (A) or guanine (G) and grown for four days. The concentrations of the purines supplemented in the agar plates are listed above or below the respective images. In addition, each of the *brsRM-*inducing transposon insertion mutations was introduced into the *brsRM-gusA* reporter strain and spotted onto chemically defined medium agar plates containing either B) no purines (–AG), C) 0.132 mM guanine (+G), D) 0.15 mM adenine (+A), or E) 0.132 mM guanine + 0.15 mM adenine (+AG). Strains are listed from left to right as: WT (parent reporter strain), 1 (*tilS* mutant), 2 (*rpoB* mutant), 3 (SMU_2060/2061 IGR insertion), 4 (*rgpD* mutant), 5 (*ssuE* mutant), 6 (SMU_1406c mutant), 7 (*prfC* mutant), 8 (SMU_1193 mutant), 9 (*mnmE* mutant), 10 (SMU_1297 mutant), and 11 (*comE* mutant). The strains were incubated for two days before imaging.

As mentioned previously, little is known about the environmental and/or cellular signals that naturally activate LRS from their basal inactive states. Given the broad distribution and conservation of LRS, it was of interest to gain further insight into LRS activation, as similar mechanisms may exist in other organisms. We created a *mariner* transposon library of >10,000 mutants to screen for mutations that could trigger activity from a transcription fusion *brsRM-gusA* β-glucuronidase reporter strain. We selected the *brsRM* LRS for several reasons: 1) we have previously studied the BrsRM LRS [29], 2) BrsRM is the most stringently regulated LRS (Fig 1B), and 3) BrsRM is the most broadly distributed LRS (Fig 4). Prior to transposon mutagenesis, we deleted all other LRS from the *brsRM-gusA* reporter strain to eliminate any potential impact of cross-regulation between LRS (Fig 1C and 1D). After screening the library, we initially identified 49 transposon mutants that exhibited various levels of β-glucuronidase activity. We retransformed these mutations into the parent *brsRM-gusA* reporter to assess reproducibility and then identified the insertion sites of clones exhibiting β-glucuronidase reporter activity (S4 Fig). The final list of 11 distinct *brsRM*-inducing mutations is shown in Table 4. We next introduced these same mutations into a *brsRM-gusA* transcription fusion reporter strain in which the *brsRM* ORFs were replaced by *gusA* (i.e. Δ*brsRM*). In the Δ*brsRM* background, only the *rgpD* and SMU_2060–2061 intergenic region (IGR) mutants still exhibited obvious reporter activity (Table 4), suggesting these two mutations increase *brsRM* operon expression independent of BrsR autoregulation (i.e. the BrsRM LRS is not required). The remaining 9 mutations in Table 4 do require BrsRM to induce reporter activity and are therefore likely to function via the activation of the BrsRM LRS. Of these, we were next interested to determine whether there is any common theme or pathway among them that might yield clues as to the source of their BrsRM activation phenotypes. After testing various hypotheses, ultimately, it was purine metabolism that proved to be a key aspect of BrsRM activation. Since several of the genes listed in Table 4 have either verified or predicted roles in purine metabolic processes (*tilS*, *mnmE*, and SMU_1297), purines were among the numerous reagents tested for *brsRM-gusA* reporter activity using chemically defined medium agar plates. As shown in Fig 6A, in adenine/guanine drop-out medium, the reporter strain exhibited no obvious response after four days of incubation. In contrast, low concentrations of adenine and guanine both served as potent activators of the reporter. Interestingly, reporter activity increased concomitantly with adenine concentration, whereas the opposite was observed with guanine (Fig 6A). We repeated the purine experiment using the mutant strains listed in Table 4 and all but the SMU_1297 mutant exhibited obvious reporter activity after incubating for only two days in the presence of adenine, and to a lesser extent, guanine as well (Fig 6B–6E). Despite the lack of reporter activity from the SMU_1297 mutant, this strain still exhibited an intriguing response to adenine, as it was the only mutant likely exhibiting adenine auxotrophy (Fig 6D and 6E). Thus, SMU_1297 is presumably an unrecognized key component of purine metabolism. Similarly, both the *rpoB* and *rgpD* mutants grew poorly on defined medium in the absence of purine supplementation, whereas both grew normally on complex medium. It is worth noting that the *rpoB* mutant likely encodes a partially functional RpoB protein, as the transposon insertion occurred near to the 3’ of the *rpoB* ORF (S4 Fig). This reduced functionality is apparently problematic for growth on chemically defined medium, as only a fraction of the *rpoB* mutant cells was able to grow in this condition (Fig 6B–6E). Despite this, the *rpoB* mutant as well as the *tilS* mutant were the only ones to exhibit obvious *brsRM* expression in the absence of purines, although purine supplementation could still further augment their reporter activity like most of the other mutants (Fig 6B–6E). Overall, these results support a major role for purines (especially adenine) as mediators of BrsRM activation.

**Table 4:** *brsRM*-inducing transposon mutations.

Strain	Function	Tn Insertion	*brsRM^*+*^	*Δ*brsRM*
Parent	*brsRM-gusA* (all other LRS deleted)	–	–	–
*tilS* (SMU_13)	tRNA(Ile)-lysidine synthase	2	++++	–
*rpoB* (SMU_1990)	RNA Polymerase beta subunit	1	+++	–
SMU_2060–2061	IGR between SMU_2060–2061 ORFs	1	+++	+++
*rgpD* (SMU_828)	Polysaccharide ABC transporter subunit	1	++	++
*ssuE* (SMU_1089)	NADPH-dependent FMN reductase	1	++	–
SMU_1406c	NADPH-dependent FMN reductase	3	++	–
*prfC* (SMU_608)	Peptide chain release factor 3	4	+	–
SMU_1193	GntR Family transcription regulator	1	+	–
*mnmE* (SMU_1235)	tRNA modification GTPase	3	+	–
SMU_1297	DHH Family phosphoesterase	2	+	–
*comE* (SMU_1917)	Sensor kinase	1	+	–

*++++ indicates strongest GusA reporter activity on complex medium agar plates

*+++ indicates strong GusA reporter activity on complex medium agar plates

*++ indicates medium GusA reporter activity on complex medium agar plates

*+ indicates weak GusA reporter activity on complex medium agar plates

## Discussion

The current study provides the first insights into a widely conserved, but almost entirely uncharacterized group of prokaryotic sensory systems. In *S*. *mutans*, these systems, termed LytTR Regulatory Systems, are included within its core genome (Table 1) and control diverse regulons as well as a cell death pathway [28, 29]. The key features of LRS are distinct from the other 2-protein sensory systems (TCSTS and ECF σ factor systems) (Fig 3A and 3B) suggesting LRS possibly represent a novel class. Despite the large number of putative LRS operons we identified amongst both bacteria and archaea, the true breadth and diversity of LRS is likely to be underestimated, as our analyses were performed using *S*. *mutans* LRS as model systems, due to the current lack of relevant studies in other species. For example, in the MRSA strain *S*. *aureus* COL, the two-gene operon SACOL_RS12395/RS12400 encodes a putative LytTR Family regulator upstream of a DUF3021-containing membrane protein and the operon contains typical LRS repeats located 9 bp upstream of the operon -35 sequence (Table 3). However, the LRS membrane protein SACOL_RS12400 lacks significant sequence similarity to those of *S*. *mutans* LRS and it exhibits a distinct predicted topology as well (S3F Fig). Despite this, the putative SACOL_RS12395/RS12400 LRS is widely encoded among the staphylococci and many other Gram positive species. A similar result can be observed from the lmo0984 –lmo0987 operon of *L*. *monocytogenes*, except this operon also includes an ABC transporter much like those associated with the SMU_433/434 and SMU_1070c/1069c LRS of *S*. *mutans* (Fig 5). Whether these LRS are weak homology orthologs of *S*. *mutans* LRS or represent entirely distinct varieties of LRS remains to be determined. However, protein topology predictions suggest the latter scenario is more likely to be the case (S3A–S3F Fig). Furthermore, we have also encountered a number of “LRS-like” operons that are analogous, but clearly distinct from those of *S*. *mutans* or the aforementioned unclassified LRS from *S*. *aureus* and *L*. *monocytogenes*. Such operons can be found among members of the *Bacteroides fragilis* group, such as *B*. *thetaiotaomicron* and *B*. *ovatus*, and exhibit a unique operon arrangement encoding transcription regulators and membrane proteins unlike those of *S*. *mutans* LRS (Table 3 and S3G Fig). Despite the unique qualities of these operons, the obvious parallels to *S*. *mutans* LRS suggest that LRS likely exist in a greater variety than is currently recognized.

One of the key features defining LRS control in *S*. *mutans* is the autoregulatory positive feedback regulation encoded within the operons. For the HdrRM LRS, this is mediated directly by HdrR and is critically dependent upon its recognition of the direct repeats located upstream of the *hdrRM* operon promoter (Fig 2A–2E). It is now evident that these direct repeats are not only key to LRS function in *S*. *mutans*, but they appear to be a defining feature of most, if not all, LRS encoded by a wide diversity of prokaryotes (Table 3). Among the putative orthologous LRS found in other species, there is low overall sequence conservation of the individual direct repeats, whereas the direct repeat lengths, their spacing, and their locations immediately upstream of LRS operon promoters are all highly conserved (Table 3). Another conserved characteristic of *S*. *mutans* LRS is the inhibitory function of LRS membrane proteins, which play key roles in dictating the basal expression levels of LRS operons (Fig 1B). Presumably, it is the inhibitory equilibrium maintained between an LRS membrane protein and its cognate regulator, which is the principal determinant of LRS operon basal expression. The inhibitory function of LRS membrane proteins can also yield misleading results when performing genetic studies of unrecognized LRS, since single mutations of LRS regulators or double mutations of cognate LRS regulators and membrane proteins are both likely to result in wild-type phenotypes [26]. To observe LRS-related phenotypes, one must solely mutate the LRS membrane protein to constitutively activate the system.

Based upon these conserved features of LRS, several inferences can be made regarding their functionality. Firstly, LRS exist in a basal inactive state. A variable, but limited amount of autoregulation is permitted under normal growth conditions (Figs 1B, 2B, 2D and 2E), which would ensure that the cell maintains a minimal abundance of LRS for the detection of relevant stimuli. Upon signal detection, LRS abundance should quickly increase due to positive feedback autoregulation, thus amplifying both the signal detection apparatus as well as the downstream transcriptional response. Secondly, LRS presumably respond to unusual growth conditions and/or environmental stress. This is supported by several observations: 1) LRS exist in a basal inactive state, 2) the HdrRM LRS responds to a rapid switch to high cell density growth conditions [26], 3) purines, which mediate activation of the BrsRM LRS (Fig 6A–6E) are also central transducers of environmental stress signals [19, 21, 22], and 4) DUF2154, which is found in HdrM, is encoded by proteins responding to cell envelope damage [40–42]. These features are also highly reminiscent of ECF systems. Like LRS, ECF systems are maintained in a basal inactive state, due to the inhibitory function of cognate anti-σ factors. Furthermore, ECF systems are similarly dispensable under normal growth conditions [43, 44] and their activation is typically dependent upon positive feedback autoregulation, ultimately triggered by environmental stress [11, 12, 15, 45]. The lack of shared domains between ECF anti-σ proteins and LRS membrane proteins (Fig 3B) as well as the obvious distinctions between σ factors and transcription regulators suggest that ECF systems and LRS evolved independently, although it is conceivable that both systems could be products of convergent evolution.

When examining the distribution of LRS, it is evident that these systems are encoded by a phylogenetically diverse group of Gram positive and Gram negative bacteria and even some archaea (Fig 4). However, their distribution appears highly biased as well with a subset of Firmicutes encoding the majority of LRS, especially the Lactic Acid Bacteria (Fig 4 and S3 Table). It is currently unclear why such a bias exists. This could be partly due to the utility of some LRS for the regulation of bacteriocin genes. Lactic Acid Bacteria are particularly rich sources of diverse bacteriocins that are regulated by LytTR Family-like repeats upstream of the bacteriocin gene -35 sequences [25, 29, 31, 46–51]. Another possibility that is not mutually exclusive with the former could be that LRS are a fairly recent evolutionary innovation originating within the Firmicutes phylum. In which case, a biased overrepresentation in these species would be expected [52]. Certainly, it is also possible, if not likely, that our current view of LRS distribution is reflective of only a subset of LRS as a consequence of our comparisons to *S*. *mutans*. In this case, an apparent skewed overrepresentation among the Lactic Acid Bacteria might be simply due to their close phylogenetic relatedness to *S*. *mutans*. As mentioned previously, the presence of LRS-like operons in other distantly related organisms hints at the possibility of a greater diversity of LRS than is currently recognized. Further clarity should arise once additional functional data are available from other LRS-encoding species.

## Materials and methods

### Bacterial species and culture conditions

All bacterial strains used in this study are listed in S1 Table and were either grown in an anaerobic chamber containing 85% N_2_, 10% CO_2_, and 5% H_2_ at 37°C, a 5% CO_2_ incubator at 37°C, or cultured with aeration at 37°C. The *S*. *mutans* strain UA140 [53] was used as the parent wild-type for all experiments. *S*. *mutans* strains were cultured using Todd Hewitt medium supplemented with 0.3% wt vol^-1^ yeast extract (THYE, Difco) or in chemically defined medium [54], while *E*. *coli* strains were cultured with Lennox LB (LB, Difco) medium. For antibiotic selection, cultures were supplemented with the following antibiotics: *S*. *mutans–*(10 μg ml^-1^ erythromycin, 1 mg ml^-1^ spectinomycin, 0.02 M *p*-chlorophenylalanine [4-CP], and 800 μg ml^-1^ kanamycin) and *E*. *coli*–(100 μg ml^-1^ ampicillin, 50 μg ml^-1^ chloramphenicol, 250 μg ml^-1^ erythromycin, and 100 μg ml^-1^ spectinomycin).

### DNA manipulation and strain construction

All primers used for strain construction are listed in S2 Table. All PCR reactions employed Phusion DNA Polymerase (NEB). PCR amplicons were purified using the Zymo Research DNA Clean & Concentrator-25. All constructs were assembled using an overlap extension PCR (OE-PCR) strategy.

### Construction of wild-type and membrane protein deletion LRS reporter strains

The *S*. *mutans* luciferase reporter strains used in Fig 1B were created by inserting the green renilla luciferase ORF immediately downstream of the LRS operons. Briefly, the luciferase open reading frame (ORF) containing the *S*. *mutans ldh* (lactate dehydrogenase) ribosome binding site was amplified from the strain ldhRenGSm [55] using the primer pair RenG-F/RenG-R. The *ermAM* erythromycin resistance cassette was PCR amplified from the plasmid pJY4164 [56] using the primer pair (RenG) erm-F/erm-R. Primers used to amplify the respective upstream and downstream homologous fragments for each reporter construct are as follows: wild-type SMU_294/295 LRS [SMU294-LF/SMU295(RenG)-R and (erm)SMU295-RF/SMU295-RR], SMU_294/Δ295 LRS [SMU294-LF/SMU294(RenG)-R and (erm)SMU294-RF/SMU295-RR], wild-type SMU_1070c/1069c LRS [SMU1070c-LF/SMU1069c(RenG)-R and (erm)SMU1069c-RF/ SMU1070c-RR], SMU_1070c/Δ1069c LRS [SMU1070c-LF/SMU1070c(RenG)-R and (erm)SMU1070c-RF/SMU1070c-RR], wild-type SMU_1854/1855 (*hdrRM*) LRS [hdrRM159-LF/hdrM(RenG)-R and (erm)hdrM-RF/hdrRM159-RR-2], SMU_1854/Δ1855 (*hdrR*Δ*M*) LRS [hdrRM159-LF/hdrR(RenG)-R and (erm)hdrR-RF/hdrRM159-RR-2], SMU_2080/2081 (*brsRM*) LRS [brsM-LF/brsM(RenG)-R and (erm)brsM-RF/brsM-RR], SMU_2080/Δ2081 (*brsR*Δ*M*) LRS [brsM-LF/brsR(RenG)-R and (erm)brsR-RF/brsM-RR]. All PCR amplicons were purified and mixed in equal molar concentrations and then subjected to a 4-fragment OE-PCR reaction using the respective upstream forward/downstream reverse primer pairs. The assembled PCR amplicons were transformed into *S*. *mutans* strain UA140 and selected on agar plates supplemented with erythromycin to obtain the following strains: 294-295-RenG, 294-RenG, 1070c-1069c-RenG, 1070c-RenG, hdrRM-RenG, hdrR-RenG, brsRM-RenG, and brsR-RenG. The wild-type SMU_433/434 and SMU_433/Δ434 LRS luciferase reporter constructs were PCR amplified from strains 01-luc and 01-luc-434. The resulting PCR amplicons were then transformed into *S*. *mutans* strain UA140 and selected on agar plates supplemented with spectinomycin to obtain the strains 433-434-RenG and 433-RenG.

### Construction of LRS deletion strains

To create markerless in-frame deletions of all 5 LRS in *S*. *mutans* UA140, we first deleted SMU_433/434 using our previously described markerless mutagenesis protocol [57]. Two fragments corresponding to the upstream and downstream regions of the SMU_433/434 operon were amplified with the primer pairs SMU433-LF/(IFDC2)smu433-LR and (IFDC2)smu434-RF/SMU434-RR, respectively. The IFDC2 cassette was amplified from the plasmid pIFDC2 [57] using the primer pair ldhF/ermR. The three fragments were mixed and used as templates for OE-PCR with the primer pair SMU433-LF/SMU434-RR. The resulting OE-PCR product was transformed into UA140 and selected on medium containing erythromycin to isolate transformants containing the IFDC2 cassette. Next, DNA fragments containing the SMU_433 upstream region and SMU_434 downstream region were amplified with the primer pairs SMU433-LF/smu433-LR2 and smu434-RF2/SMU434-RR. The two fragments were mixed and assembled with OE-PCR using the primer pair SMU433-LF/SMU434-RR. The OE-PCR amplicon was then transformed into the IFDC2-containing strain and selected on the medium containing *p*-chlorophenylalanine (4-CP) to remove the IFDC2 cassette and obtain the markerless deletion mutant. This strain was then used as a recipient for the sequential deletion of SMU_1070c/1069c, SMU_294/295, *hdrRM*, and *brsRM* using the same approach to obtain the final 5 LRS deletion strain ifdLRS.

### Construction of single LRS luciferase reporter strains

Genomic DNA from strains 294-295-RenG, 1070c-1069c-RenG, hdrRM-RenG, brsRM-RenG, and 433-434-RenG were transformed into strain ifdLRS and selected on THYE plates contains erythromycin or spectinomycin to obtain the single LRS luciferase reporter strains ifdLRS/294-295-RenG, ifdLRS/1070c-69c-RenG, ifdLRS/hdrRM-RenG, ifdLRS/brsRM-RenG, and ifdLRS/433-434-RenG.

To examine potential cross-regulation between different LRS, ORFs encoding LRS membrane proteins were replaced by a kanamycin resistance cassette using the single LRS luciferase reporter strains as recipients. Briefly, upstream and downstream homologous fragments of SMU_295 were amplified using the primer pairs SMU294-LF/(kan)smu295-LR and (kan)smu295-RF/SMU295-RR as well as UA140 genomic DNA as a template. The kanamycin resistance gene was amplified using the primer pair kan-F/kan-R and plasmid pWVTKs [58] as the template. Three fragments were mixed and assembled with OE-PCR using the primer pair SMU294-LF/SMU295-RR. The OE-PCR amplicon was transformed into the single luciferase reporter strains ifdLRS/1070c-69c-RenG, ifdLRS/hdrRM-RenG, ifdLRS/brsRM-RenG and ifdLRS/433-434-RenG to obtain d295/1070c-69c-RenG, d295/hdrRM-RenG, d295/brsRM-RenG and d295/433-434-RenG. A similar approach was used to delete *hdrM* and *brsM* in each of the single LRS reporter strains. The SMU_434 and SMU_1069c mutations were PCR amplified from d-smu434/UA140 and d-smu1069/UA140 and then transformed into the single LRS reporter strains.

### Creation of *hdrRM* luciferase reporter strains for promoter analyses

The *S*. *mutans* firefly luciferase reporter strains used in Fig 2 were created using a markerless mutagenesis approach. To create the markerless replacement of the *hdrRM* ORFs with that of luciferase, we first created an allelic replacement of the *hdrRM* ORFs with the counterselectable IFDC2 cassette [57]. Using UA140 genomic DNA as a template, two fragments corresponding to the upstream and downstream regions of the *hdrRM* operon were amplified with the primer pairs hdrRupF/hdrRupR-ldh and hdrMdnF-erm/hdrMdnR, respectively. The IFDC2 cassette was amplified using the primer pair ldhF/ermR. The three fragments were mixed and used as template for OE-PCR with the primer pair hdrRupF/hdrMdnR. The resulting OE-PCR product was transformed into UA140 and selected on medium containing erythromycin to obtain strain RMIFDC2. Next, a DNA fragment containing the *hdrR* upstream region and firefly luciferase ORF was amplified with the primer pair hdrRupF/lucR-1856 and strain LZ89-luc [26] as a template. Using strain UA140 as a template, a fragment corresponding to the *hdrM* downstream region was amplified with the primer pair 1856F-luc/hdrMDnR. The two fragments were mixed and assembled with OE-PCR using the primer pair hdrRupF/hdrMdnR. The OE-PCR amplicon was transformed into strain RMIFDC2 and selected on medium containing *p-*chlorophenylalanine (4-CP) to obtain strain RpLuc. To create strains Rp+1luc and Rp-10mluc, the upstream and downstream regions of the *hdrRM* operon were amplified from strain UA140 with the primer pairs hdrRupF/(luc)hdrRp-R or hdrRupF/(luc)hdrRp-10-R and (lucR)hdrMdn-F/hdrMDn-R, respectively. The luciferase ORF was amplified from strain RpLuc with the primer pair lucF/lucR. The three fragments were mixed and used as template for OE-PCR with the primer pair hdrRupF/hdrMdnR. OE-PCR products were transformed into RMIFDC2 and selected on medium containing 4-CP to obtain the strains Rp+1luc and Rp-10mluc. Strains Rp+1luc and Rp-10mluc were both transformed with the plasmid pHdrRoe [27] to create the strains Rp+1lucROE and Rp+1lucROE-10. Using the genomic DNA from strain RpLuc as a template, two fragments were amplified with the primer pairs hdrRupF/(repeat-m)hdrR-LR and (repeat-m)hdrR-RF/hdrMDnR. The two PCR amplicons were mixed with hybridized EMSA-hdrRpm-F/R primers and assembled using OE-PCR with the primer pair hdrRupF/hdrMdnR. The OE-PCR amplicon was transformed into strain RMIFDC2 and selected on medium containing 4-CP to create the strain RpDRmluc. To create the *hdrR* ectopic overexpression plasmid pJYROE, a fragment containing the *hdrR* ORF fused to the *ldh* promoter was first amplified from pHdrRoe using the primer pair ldhF-bamHI/hdrRR-hindIII. The resulting PCR amplicon was digested with *Bam*HI and *Hin*dIII and then ligated to pJY4164 to obtain the suicide vector pJYROE. To create the *hdrM* ectopic overexpression plasmid pMOE, an *ldh* promoter-*hdrM* transcription fusion was assembled by first PCR amplifying the *ldh* promoter and *hdrM* ORF using the primer pairs ldhF-BamHI/ldhR-SpeI and hdrMF-SpeI/hdrMR-EcoRI as well as UA140 gDNA as a template. The resulting amplicons were then digested with *Bam*HI/*Spe*I and *Spe*I/*Eco*RI and subsequently ligated to the *Bam*HI/*Eco*RI restriction sites of the *E*. *coli*-*Streptococcus* shuttle vector pDL278 [59] to create the plasmid pMOE. The suicide vector pJYROE was transformed into strain RpLuc or RpDRmluc to create the strains ROE or ROE/DR-, while the shuttle vector pMOE was transformed into strain ROE to obtain the strain RMOE.

To insert the luciferase ORF downstream of the *hdrRM* ORFs, a DNA fragment containing the *hdrR* upstream region and IFDC2 were PCR amplified from strain RMIFDC2 with the primer pair hdrRupF/ermR-lucf. Using the genomic DNA of RpLuc as a template, the luciferase ORF was amplified with the primer pair lucF-erm/lucmR. The two amplicons were assembled using OE-PCR and the primer pair hdrRupF/lucmR. The resulting overlapping PCR products were transformed into RpLuc strain and selected on medium containing erythromycin to obtain the strain RMlucIFDC2. Next, two fragments encompassing the *hdrRM* locus were amplified from strain UA140 with the primer pair hdrRupF/MterR-luc, while the luciferase ORF was amplified from strain RpLuc with the primer pair lucF-Mter/lucmR. The PCR amplicons were mixed and assembled by OE-PCR using the primer pair hdrRupF/lucmR. The resulting OE-PCR amplicon was transformed into strain RMlucIFDC2 and selected on plates supplemented with 4-CP to obtain the strain hdrRMluc. To mutate *hdrM* in strain hdrRMluc, three fragments were amplified from this strain using the primer pairs hdrRupF/(spec)smu1853R, (spec)smu1853-hdrR-LF2/hdrM(TAA)R, and hdrM(TAA)F/lucmR. The spectinomycin resistance cassette *aad9* was amplified from the *E*. *coli-Streptococcus* shuttle vector pDL278 [59] using the primer pair specF/specR. The four amplicons were mixed and assembled by OE-PCR using the primer pair hdrRupF/lucmR. The resulting OE-PCR amplicon was transformed into strain hdrRMluc to obtain the strain dhdrMluc. To mutate the direct repeats upstream of the *hdrRM* promoter in strain dhdrMluc, two fragments were amplified from this strain using the primer pair hdrRupF/(repeat-m)hdrR-LR and (repeat-m)hdrR-RF/lucmR. The two PCR amplicons were mixed with hybridized EMSA-hdrRpm F/R primers and assembled using OE-PCR and the primers hdrRupF/lucmR. The resulting OE-PCR amplicon was transformed into strain hdrRMluc to obtain the strain dhdrMdDRluc.

### Construction of *brsRM*-*gusA* reporter strains

To create markerless *gusA* transcription fusions to the *brsRM* operon, a *brsRM* upstream homologous fragment was amplified from strain UA140 or ifdLRS using the primer pair brsRM-LF/(gusA)brsRM-LR, while the *brsRM* downstream homologous fragment was amplified from strain UA140 using the primer pair (gusA)brsRM-RF/brsRM-RR. The *gusA* ORF was amplified from plasmid pZX7 [60] using the primer pair GusA-F/GusA-R. The three amplicons were assembled via OE-PCR with the primer pair brsRM-LF/brsRM-RR. The two resulting OE-PCR amplicons were then transformed into the strain ifdLRS/brsRM(IFDC2) and selected on the medium containing 4-CP to obtain the strains ifdLRS/brsRM-gusA and ifdLRS/brsRMp-gusA respectively.

### Generation of a transposon insertion library in the *brsRM-gusA* reporter strain

The ifdLRS/brsRM-gusA reporter strain transposon library was generated by a previously described transposon mutagenesis protocol [61]. Briefly, the primer pair MmeI-MGL-erm-F/MmeI-MGL-erm-R was used to amplify the erythromycin resistance cassette from plasmid pJY4164. Sequences at the 5’ ends of both primers add repeat sequences recognized by the *himar* transposon onto both ends of the PCR amplicon. The resulting amplicon was then ligated to the pGEM^®^-T vector (Promega) to obtain pT-MGL-erm. *In vitro* transposon mutagenesis was performed by combining MarC9 transposase, genomic DNA from strain ifdLRS, and plasmid pT-MGL-erm and then incubating at 30°C for 1 h. Transposon junctions were subsequently repaired and then the transposition reaction was transformed into strain ifdLRS/brsRM-gusA. Transposon mutants were selected on THYE plates containing erythromycin and 5-bromo-4-chloro-3-indolyl-β-D-glucuronic acid (X-gluc, 200 μg ml^-1^). After 5 days of incubation, blue colonies were selected. Transposon insertion sites were mapped according to the published protocol [61], except that PCR amplicons were ligated into the pGEM^®^-T vector, transformed into *E*.*coli* DH5α, and then the resulting plasmid inserts were sequenced. PCR was used to confirm the expected locations of transposon insertions sites in each of the mutant strains. Genomic DNA from confirmed transposon mutants was also transformed into strain ifdLRS/brsRMp-gusA (Δ*brsRM*) to compare its reporter activity with the corresponding transposon mutants obtained in the ifdLRS/brsRM-gusA (*brsRM*+) background.

### Creation of the *hdrR* recombinant expression vector

The *hdrR* ORF was amplified from strain UA140 using the primer pair hdrRF-NdeI/HdrRR-Hind. The amplicon was then digested with *Nde*I/*Hin*dIII and ligated to the expression vector pET29b to create the plasmid pEcROE.

### Recombinant protein expression and purification

Recombinant HdrR was purified using pET29b and the *E*. *coli* BL21(DE3) pLysS expression system. Cultures were grown to OD_600_ 0.6 at 37°C with aeration before adding 0.1 mM IPTG and culturing for an additional 12 hr. at 20°C. Cells were harvested by centrifugation (6000 x g, 5 min, 4°C), washed twice with binding buffer (20 mM Tris, 300 mM NaCl, 5 mM imidazole, 10% glycerol, pH 7.9) and then resuspended in 20 ml of the same buffer. Next, the cells were chilled on ice, lysed by sonication, centrifuged to recover supernatants (20,130 x g, 20 min, 4°C), and then HdrR-His6 was purified using Ni-NTA agarose chromatography (Novagen). Proteins were eluted with 4 ml elution buffer (20 mM Tris, 300 mM NaCl, 500 mM imidazole, 10% glycerol, pH 7.9) and concentrated by ultrafiltration (Millipore membrane, 3 kDa cut-off size). Purified proteins were stored in 10% glycerol at -80°C.

### Electrophoretic mobility shift assays (EMSA)

EMSAs were performed similarly as previously described [62]. Briefly, double-stranded probes were obtained by annealing equal molar concentrations of two oligonucleotides (S2 Table) in 50 mM Tris-HCl (pH 8.0), 10 mM MgCl_2_, 50 mM NaCl and 1 mM EDTA, with the forward primer 5′-end labeled with digoxigenin-11-ddUTP (Roche). The oligonucleotide pair EMSA-hdrRp-F/EMSA-hdrRp-R served as the wild-type probe, while the oligonucleotide pair EMSA-hdrRpm-F/EMSA-hdrRpm-R served as the direct repeat mutant probe. 1 ng of DNA probe was incubated individually with various concentrations of HdrR-His_6_ at 25°C for 20 min in a 20 μl reaction volume. After incubation, the reaction mixtures were separated by electrophoresis and electro-transferred to nylon membranes. Images were detected using chemiluminescence and X-ray films. For competition experiments, 50- and 200-fold excess of unlabeled probes (S2 Table) were added to the binding reactions before performing electrophoresis and imaging as described above.

### Luciferase assays

Assays of firefly and green renilla luciferase activity were performed using a previously described methodology [55] with mid-log phase cultures. Reporter data were normalized by dividing luciferase values by their corresponding optical density (OD_600_) values. Luciferase activity was measured with a GloMax Discover 96-well luminometer (Promega).

### Identification of putative LRS in other species

To identify homologs of LRS membrane proteins, we searched the NCBI non-redundant nucleotide collection (nr/nt) and whole-genome shotgun (wgs) databases using tBLASTn (E-value <10, >25% positives). These putative LRS membrane proteins (except for SMU_295 homologs) were then refined contingent on containing either DUF3021 or DUF2154 domains, as determined by NCBI RPS-tBLASTn (E-value <1). Qualifying LRS membrane protein results were further filtered based upon the presence of adjacent upstream LytTR Family transcription regulator homologs identified using tBLASTn (E-value <0.1).

### Assay for purine stimulation of *brsRM-gusA* expression

To assess the effect of purines on the BrsRM LRS, overnight cultures of ifdLRS/brsRM-gusA and isogenic transposon mutants were harvested by centrifugation, washed thrice with an equal volume of 0.9% NaCl, and spotted on adenine/guanine-replete or adenine/guanine drop-out chemically defined medium (CDM) agar plates [54]. Different concentrations of adenine (0 mM, 0.075 mM, 0.15 mM, 0.3 mM and 0.6 mM) or guanine (0 mM, 0.066 mM, 0.132 mM, 0.264 mM and 0.53 mM) were added to the CDM medium and plates were incubated at 37°C with 5% CO_2_ for 4 days. To assay the impact of purines on the transposon mutants of ifdLRS/brsRM-gusA, adenine and/or guanine was added to the CDM at a final concentration of 0.15 mM and/or 0.132 mM, respectively. Plates were incubated at 37°C with 5% CO_2_ for 2.5 days.

### Statistical analysis

All statistical analyses were performed using GraphPad Prism software to calculate significance via two-tailed Student’s *t-*tests with Welch’s correction. Statistical significance was assessed using a cutoff value of P < 0.05.

## Supporting information

S1 Fig Comparison of hdrRM luciferase reporter strains. The specific activities of the reporter strains described in Fig 2 of the text are shown for a direct comparison of their expression characteristics. The dashed red line indicates the average background luminescence measured in the assay. The blue bars represent strains listed in Fig 2B. For these reporters, the chromosomal copy of the *hdrRM* operon was replaced by a luciferase ORF, which was fused to the operon transcriptional start site (+1). For strain R^OE^, *hdrR* was ectopically expressed from a constitutive promoter on a multicopy plasmid. The orange bars correspond to the strains listed in Fig 2D. The reporters all have a luciferase ORF transcriptionally fused immediately downstream of the *hdrRM* ORFs. The green bars correspond to the strains listed in Fig 2E. These reporters have the chromosomal copy of the *hdrRM* ORFs replaced by that of luciferase. For strain RM^OE^, the *hdrR* ORF was ectopically expressed in a single copy on the chromosome using a constitutive promoter, while the *hdrM* ORF was ectopically expressed from a constitutive promoter on a multicopy plasmid. Luciferase data are expressed as means ± s.d. (indicated by error bars) derived from four biological replicates.
(TIF)

S2 Fig Comparison of conserved residues in S. mutans response regulators vs. LRS regulators. A) Clustal Omega was used to align the *S*. *mutans* LytTR Family response regulators ComE and LytR along with the well characterized response regulators VicR and CiaR. Residues marked with an asterisk indicate conserved residues. The residues shown in red font represent the conserved aspartate residues that are the sites of phosphorylation from cognate sensor kinases. B) Clustal Omega was used to align the five *S*. *mutans* LRS regulators. Residues marked with an asterisk indicate conserved residues.
(TIF)

S3 Fig Comparison of LRS membrane protein topologies. Protter [36, 37] was used to illustrate the protein topologies of each *S*. *mutans* LRS membrane protein as well as putative LRS membrane proteins from other species. For A-E, the predicted protein topology of each *S*. *mutans* LRS membrane protein was compared to its corresponding weakest similarity protein shown in Fig 5 of the text. Genes are listed by their NCBI Gene Locus Tags, while the BLASTP E-values of the two proteins are shown in parentheses. A) Comparison of SMU_295 with CSX00_RS10965 from *Pseudobutyrivibrio ruminis* (E-value e = 1.4 x 10^−10^). B) Comparison of SMU_433 with OEOE_0725 from *Oenococcus oeni* (E-value e = 4.2 x 10^−7^). C) Comparison of SMU_1069c with BUB90_RS22585 from *Anaerosporobacter mobilis* (E-value e = 2.2 x 10^−6^). D) Comparison of SMU_1855 (HdrM) with ERS095036_10318 from *Chlamydia trachomatis* (E-value e = 9 x 10^−6^). Residues shown in red represent a putative cleavable signal sequence. E) Comparison of SMU_2081 (BrsM) with TALC_RS05575 from the Thermoplasmatales archaeon BRNA1 (E-value e = 1 x 10^0^). F) Predicted topology of SACOL_RS12400 from *Staphylococcus aureus*. Residues shown in red represent a putative cleavable signal sequence. G) Predicted topology of Btheta7330_RS19920 from *Bacteroides thetaiotaomicron*.
(TIF)

S4 Fig Insertion sites of brsRM-activating transposon mutations. Red arrows mark the locations of transposon insertions resulting in activation of the *brsRM-gusA* reporter strain. Open reading frames are drawn to scale. Note: two identical, but independent *tilS* transposon insertion mutants were isolated.
(TIF)

S1 Table Strains and plasmids used in this study. *Em—erythromycin; Sp—spectinomycin; Km—kanamycin; Cm—chloramphenicol; 4-CP—p-chlorophenylalanine.
(XLSX)

S2 Table Primers used in this study. (XLSX)

S3 Table Global analysis of prokaryotic LRS operons. (XLSX)
