# Supplementary figures and images for: LytTR Regulatory Systems: A potential new class of prokaryotic sensory system
Source: PLoS Genet. 2018 Oct 8;14(10):e1007709. doi: 10.1371/journal.pgen.1007709 (PMC6193735; doi:10.1371/journal.pgen.1007709)

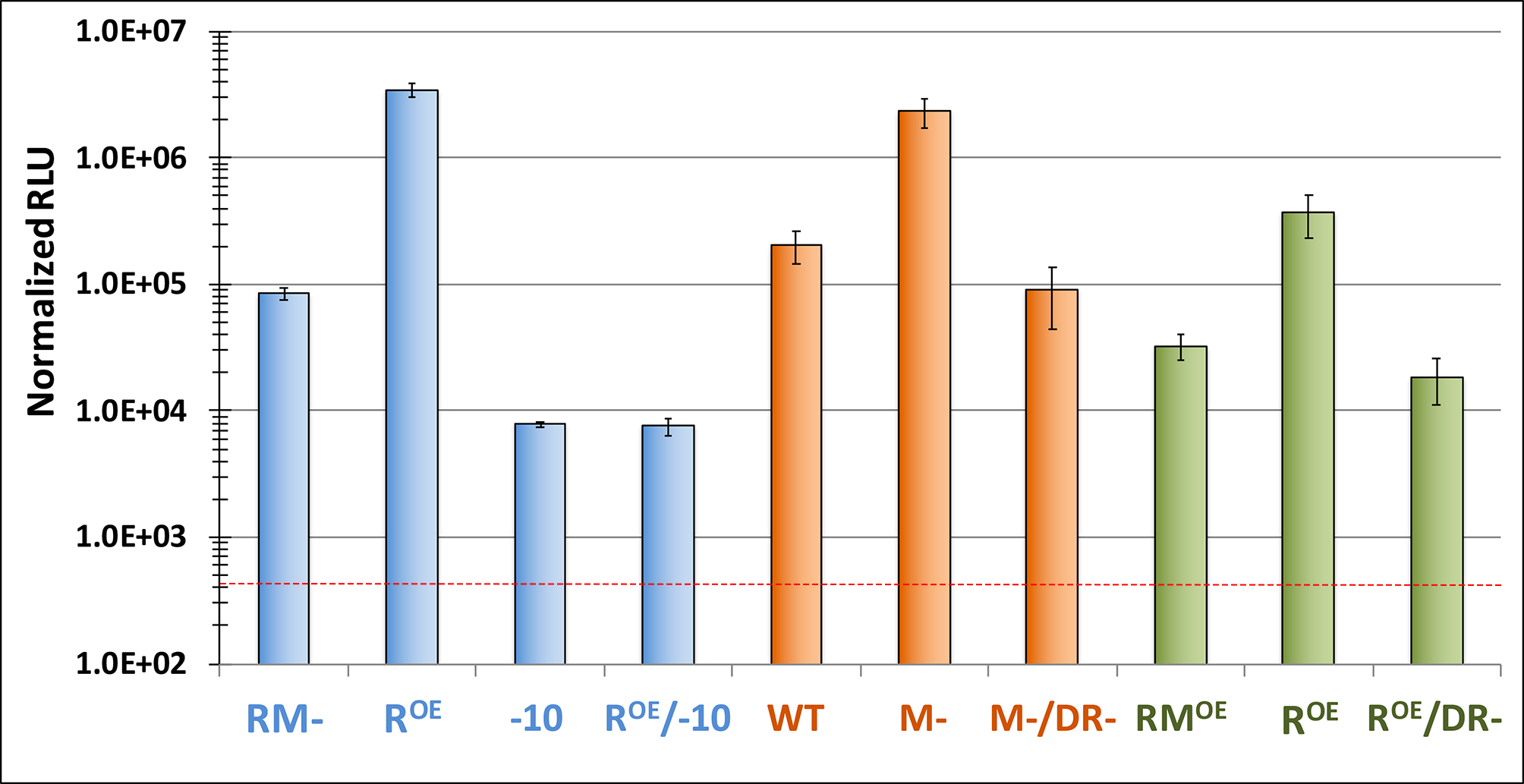

Supplement: S1 Fig — The specific activities of the reporter strains described in Fig 2 of the text are shown for a direct comparison of their expression characteristics. The dashed red line indicates the average background luminescence measured in the assay. The blue bars represent strains listed in Fig 2B. For these reporters, the chromosomal copy of the hdrRM operon was replaced by a luciferase ORF, which was fused to the operon transcriptional start site (+1). For strain ROE, hdrR was ectopically expressed from a constitutive promoter on a multicopy plasmid. The orange bars correspond to the strains listed in Fig 2D. The reporters all have a luciferase ORF transcriptionally fused immediately downstream of the hdrRM ORFs. The green bars correspond to the strains listed in Fig 2E. These reporters have the chromosomal copy of the hdrRM ORFs replaced by that of luciferase. For strain RMOE, the hdrR ORF was ectopically expressed in a single copy on the chromosome using a constitutive promoter, while the hdrM ORF was ectopically expressed from a constitutive promoter on a multicopy plasmid. Luciferase data are expressed as means ± s.d. (indicated by error bars) derived from four biological replicates. (TIF) [file pgen.1007709.s001.tif]

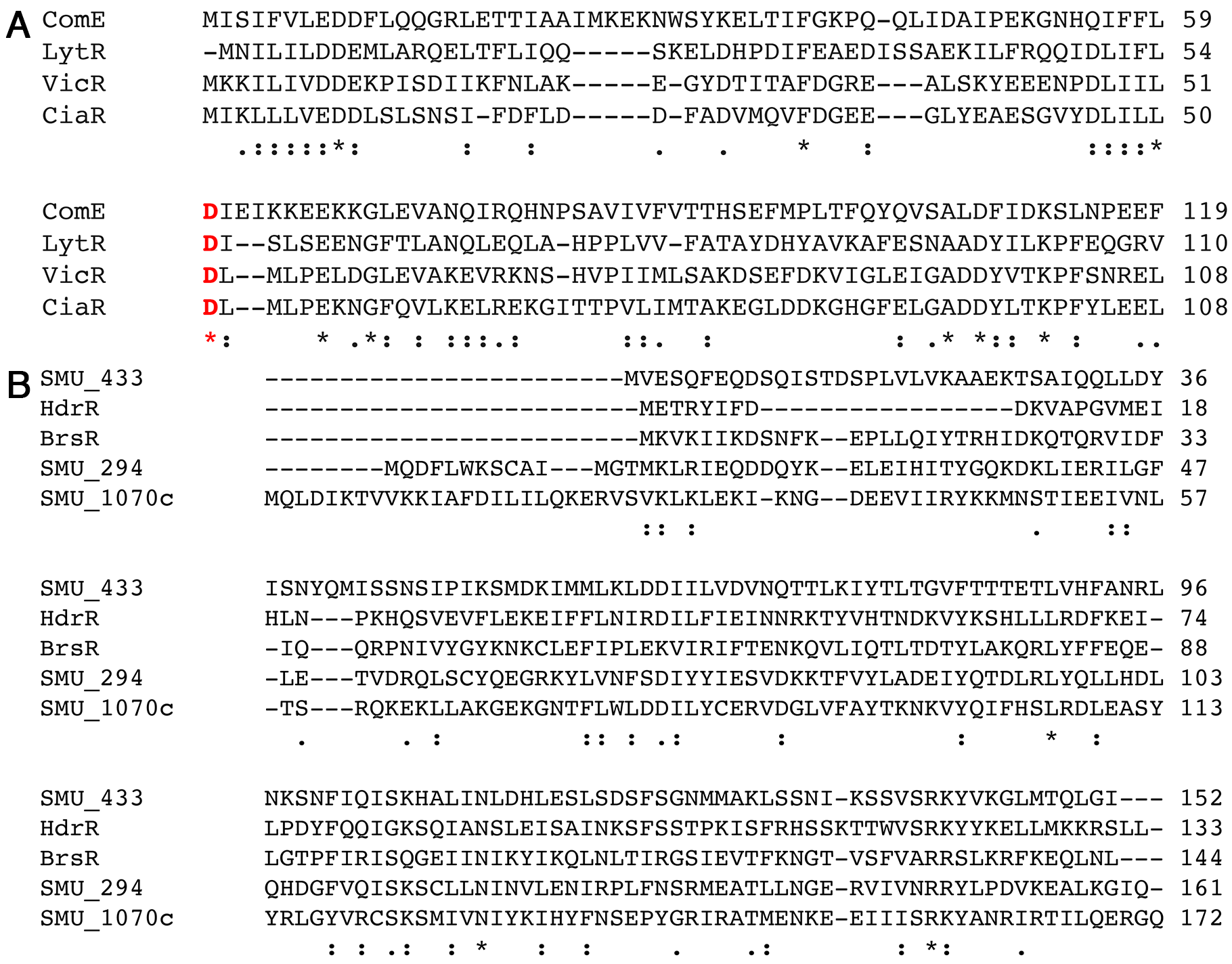

Supplement: S2 Fig — A) Clustal Omega was used to align the S. mutans LytTR Family response regulators ComE and LytR along with the well characterized response regulators VicR and CiaR. Residues marked with an asterisk indicate conserved residues. The residues shown in red font represent the conserved aspartate residues that are the sites of phosphorylation from cognate sensor kinases. B) Clustal Omega was used to align the five S. mutans LRS regulators. Residues marked with an asterisk indicate conserved residues. (TIF) [file pgen.1007709.s002.tif]

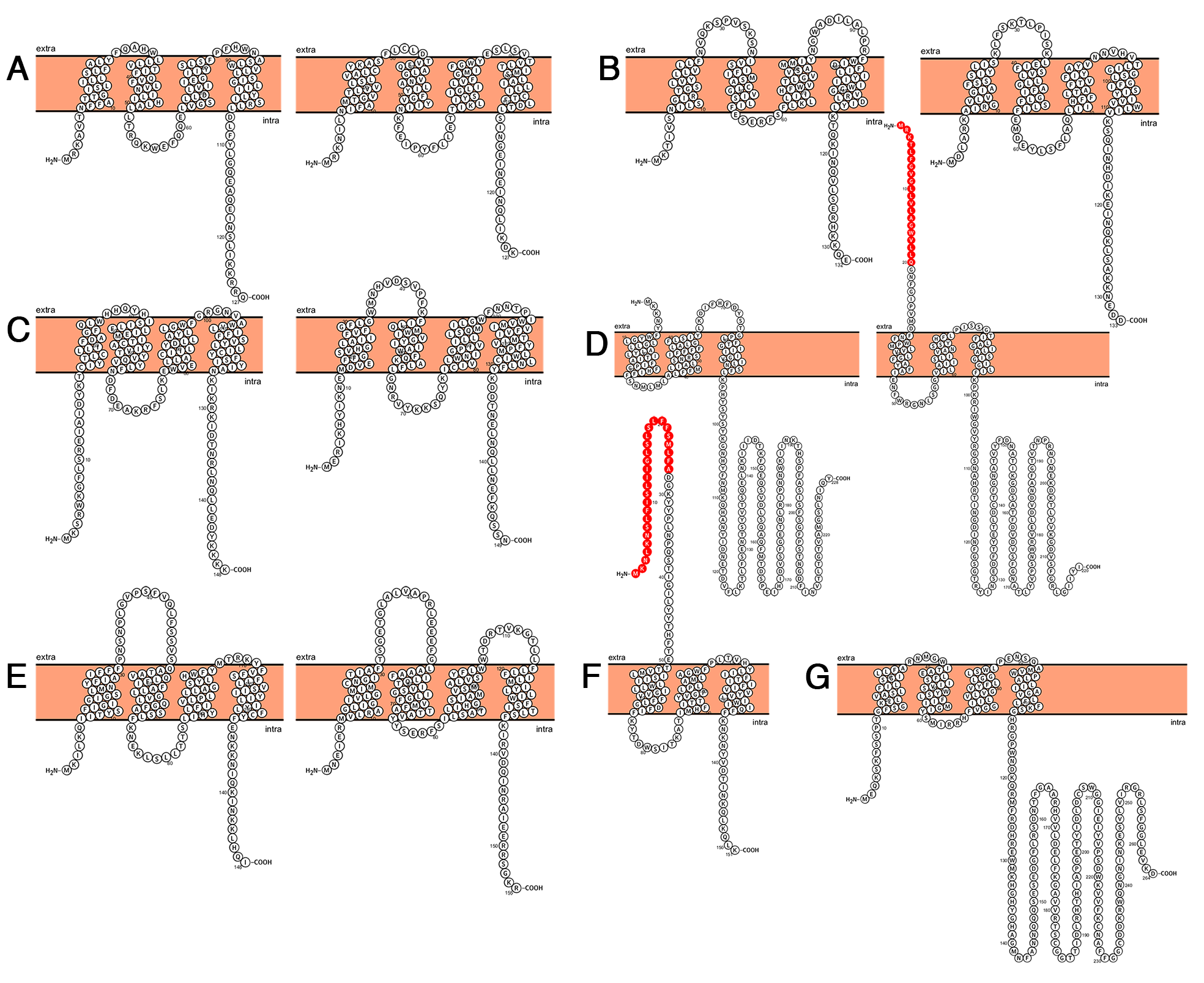

Supplement: S3 Fig — Protter [36, 37] was used to illustrate the protein topologies of each S. mutans LRS membrane protein as well as putative LRS membrane proteins from other species. For A-E, the predicted protein topology of each S. mutans LRS membrane protein was compared to its corresponding weakest similarity protein shown in Fig 5 of the text. Genes are listed by their NCBI Gene Locus Tags, while the BLASTP E-values of the two proteins are shown in parentheses. A) Comparison of SMU_295 with CSX00_RS10965 from Pseudobutyrivibrio ruminis (E-value e = 1.4 x 10^−10^). B) Comparison of SMU_433 with OEOE_0725 from Oenococcus oeni (E-value e = 4.2 x 10^−7^). C) Comparison of SMU_1069c with BUB90_RS22585 from Anaerosporobacter mobilis (E-value e = 2.2 x 10^−6^). D) Comparison of SMU_1855 (HdrM) with ERS095036_10318 from Chlamydia trachomatis (E-value e = 9 x 10^−6^). Residues shown in red represent a putative cleavable signal sequence. E) Comparison of SMU_2081 (BrsM) with TALC_RS05575 from the Thermoplasmatales archaeon BRNA1 (E-value e = 1 x 100). F) Predicted topology of SACOL_RS12400 from Staphylococcus aureus. Residues shown in red represent a putative cleavable signal sequence. G) Predicted topology of Btheta7330_RS19920 from Bacteroides thetaiotaomicron. (TIF) [file pgen.1007709.s003.tif]

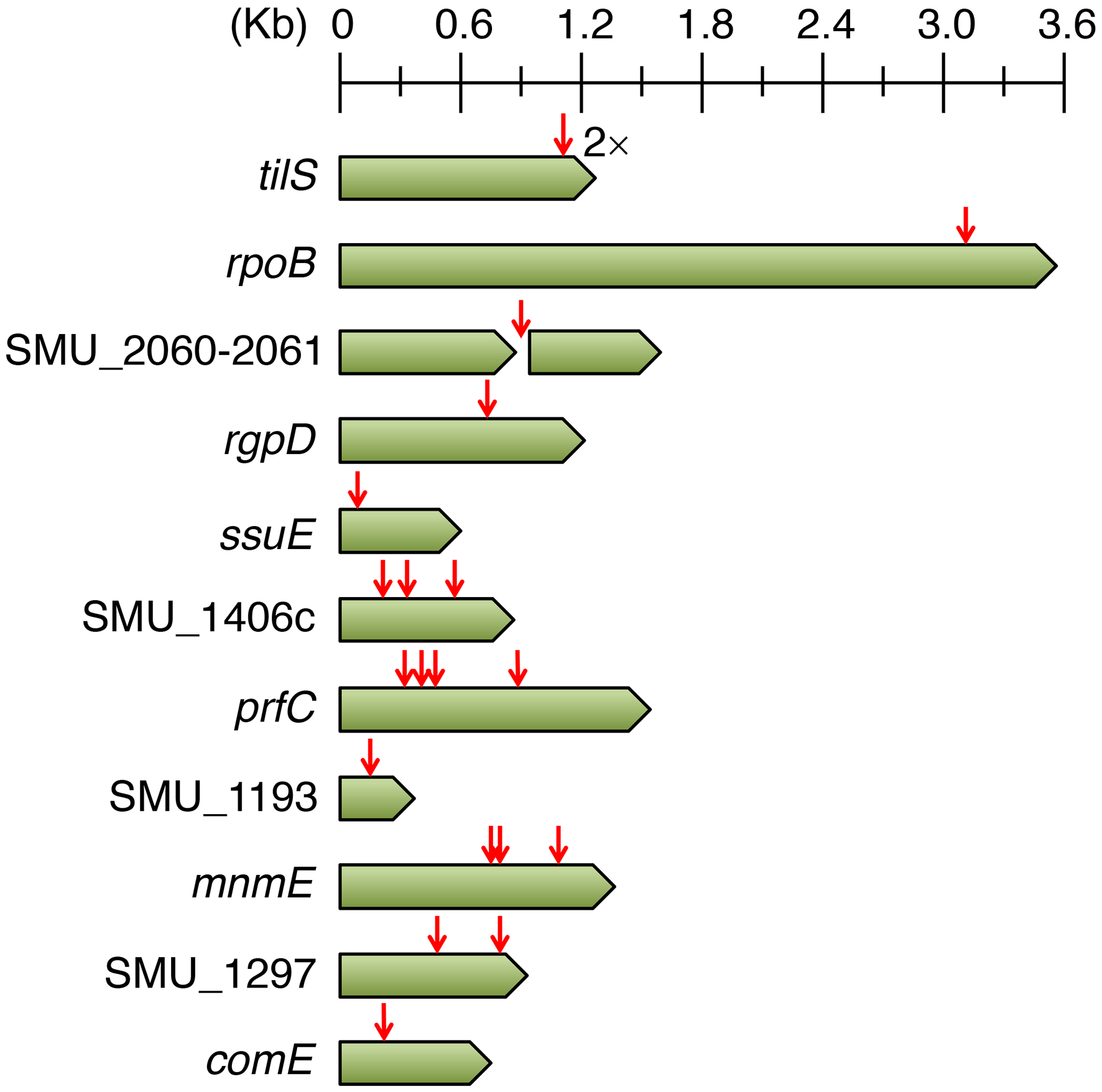

Supplement: S4 Fig — Red arrows mark the locations of transposon insertions resulting in activation of the brsRM-gusA reporter strain. Open reading frames are drawn to scale. Note: two identical, but independent tilS transposon insertion mutants were isolated. (TIF) [file pgen.1007709.s004.tif]
